# Supplementary material for: IL-22 initiates an IL-18-dependent epithelial response circuit to enforce intestinal host defence
Source: Nat Commun. 2022 Feb 15;13:874. doi: 10.1038/s41467-022-28478-3 (PMC8847568; doi:10.1038/s41467-022-28478-3)
Supplement: Supplementary file 1 — Supplementary information [file 41467_2022_28478_MOESM1_ESM.pdf]

IL-22 initiates an IL-18-dependent epithelial response circuit to enforce intestinal host defence

Supplementary Information

Chiang, *et al.*

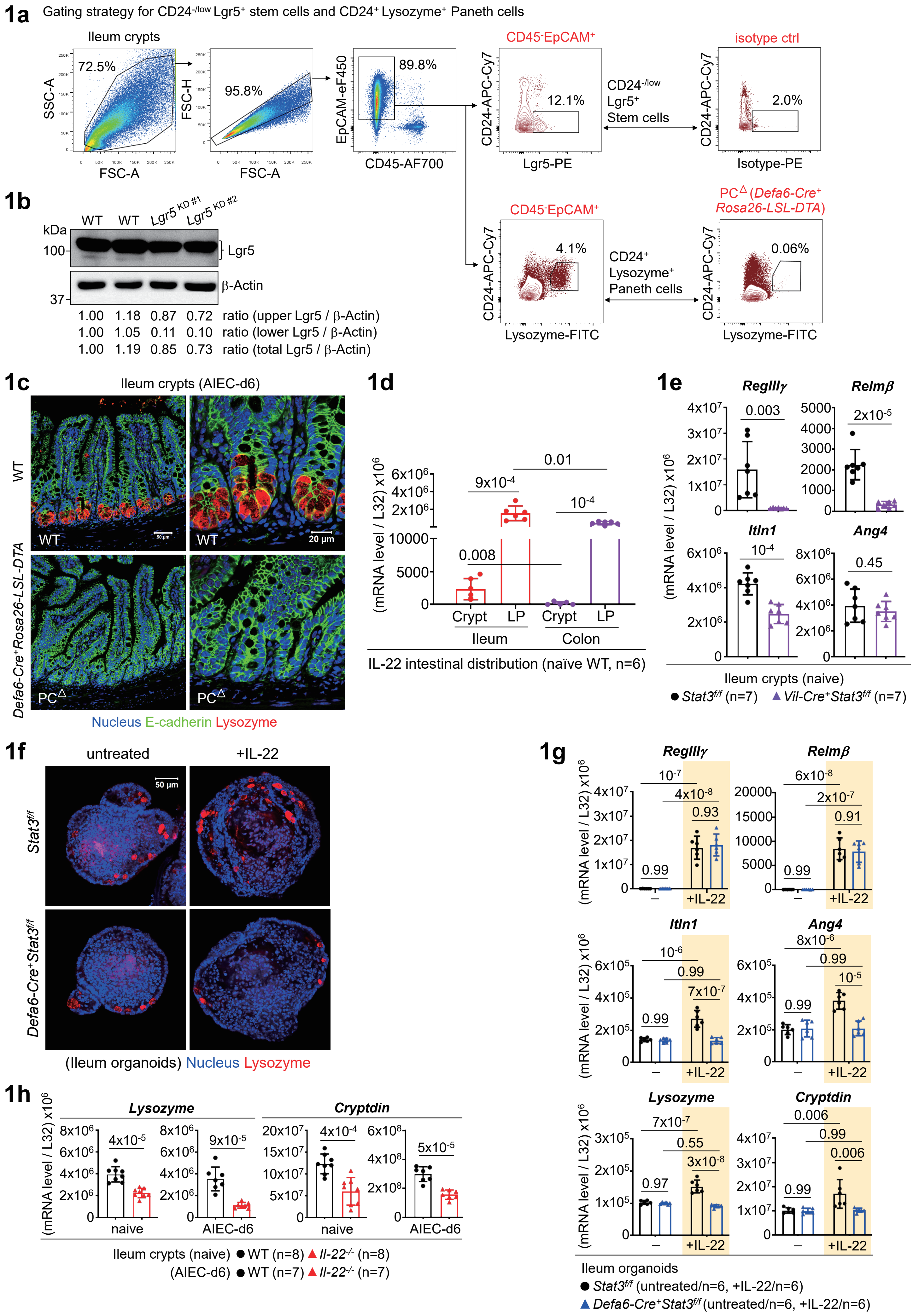
1a
Gating strategy for CD24-/low Lgr5+ stem cells and CD24+ Lysozyme+ Paneth cells
Ileum crypts
72.5%
SSC-A
FSC-A
95.8%
FSC-H
FSC-A
89.8%
EpCAM-eF450
CD45-AF700
CD45-EpCAM+
12.1%
CD24-APC-Cy7
Lgr5-PE
CD24-/low Lgr5+ Stem cells
isotype ctrl
2.0%
CD24-APC-Cy7
Isotype-PE
CD45-EpCAM+
4.1%
CD24-APC-Cy7
Lysozyme-FITC
CD24+ Lysozyme+ Paneth cells
PCΔ (Defa6-Cre+ Rosa26-LSL-DTA)
0.06%
CD24-APC-Cy7
Lysozyme-FITC
1b
kDa
WT WT Lgr5 KD #1 Lgr5 KD #2
100
37
Lgr5
β-Actin
1.00 1.18 0.87 0.72 ratio (upper Lgr5 / β-Actin)
1.00 1.05 0.11 0.10 ratio (lower Lgr5 / β-Actin)
1.00 1.19 0.85 0.73 ratio (total Lgr5 / β-Actin)
1c
Ileum crypts (AIEC-d6)
WT
Defa6-Cre+Rosa26-LSL-DTA
WT
WT
50 μm
20 μm
PCΔ
PCΔ
Nucleus E-cadherin Lysozyme
1d
(mRNA level / L32) x10^6
6x10^6
4x10^6
2x10^6
10000
5000
0
9x10^-4
0.01
10^-4
0.008
Crypt LP Crypt LP
Ileum Colon
IL-22 intestinal distribution (naïve WT, n=6)
1e
RegIIIγ
Relmβ
0.003
2x10^-5
Itln1
Ang4
10^-4
0.45
(mRNA level / L32) x10^6
Ileum crypts (naive)
● Stat3f/f (n=7) ▲ Vil-Cre+Stat3f/f (n=7)
1f
untreated
+IL-22
50 μm
Stat3f/f
Defa6-Cre+Stat3f/f
(Ileum organoids) Nucleus Lysozyme
1g
RegIIIγ
10^-7
4x10^-8
0.93
0.99
Relmβ
6x10^-8
2x10^-7
0.91
0.99
Itln1
10^-6
0.99
7x10^-7
0.99
Ang4
8x10^-6
0.99
10^-5
0.99
Lysozyme
7x10^-7
0.55
3x10^-8
0.97
Cryptdin
0.006
0.99
0.006
0.99
(mRNA level / L32) x10^6
– +IL-22
Ileum organoids
● Stat3f/f (untreated/n=6, +IL-22/n=6)
▲ Defa6-Cre+Stat3f/f (untreated/n=6, +IL-22/n=6)
1h
Lysozyme
Cryptdin
(mRNA level / L32) x10^6
4x10^-5
9x10^-5
4x10^-4
5x10^-5
naive AIEC-d6 naive AIEC-d6
Ileum crypts (naive) ● WT (n=8) ▲ Il-22-/- (n=8)
(AIEC-d6) ● WT (n=7) ▲ Il-22-/- (n=7)

Supplementary Fig. 1. IL-22 promotes Paneth cells.

(a) Gating strategy for flow cytometry analysis of ileum crypts for $CD24^{+}$ $Lysozyme^{+}$ Paneth cells and $CD24^{-/low}$ $Lgr5^{+}$ stem cells. The gating panels here correspond to FACS data panels in the manuscript figures 1d, 1g, 2g, 3g, 4g, 4l, 7a, 7b, 7c, 8a, and in the supplementary figures 2f, 4e, and 4k. **(b)** Western blot analysis of colon epithelial CMT93 cells for Lgr5 expression in wild-type or Lgr5 knockdown ($Lgr5^{KD}$) cells. Quantification and the ratio (Lgr5 to β-actin) of protein bands are indicated. **(c)** Immunofluorescence analysis of ileum crypts for $Lysozyme^{+}$ Paneth cells at the crypt base in Paneth cell-deficient (PC^{Δ}) *Defa6-Cre⁺Rosa26-LSL-DTA* mice at the day-6 (d6) post AIEC infection. **(d)** Quantitative real-time PCR analysis of crypts or lamina propria (LP) mononuclear cells, isolated from ileum or colon tissues, for *Il-22* mRNA levels. **(e)** Quantitative real-time PCR analysis of ileum crypts for anti-microbial peptides in naïve *$Stat3^{f/f}$* vs *Vil-$Cre^{+}Stat3^{f/f}$* mice. **(f)** Immunofluorescence analysis of IL-22-stimulated ileum organoids, derived from the indicated mice, for $Lysozyme^{+}$ Paneth cells at day-5 of culture. **(g)** Quantitative real-time PCR analysis of IL-22-stimulated ileum organoids, derived from the indicated mice, for anti-microbial peptides. **(h)** Quantitative real-time PCR analysis of ileum crypts, derived from the indicated uninfected or AIEC-infected mice, for Paneth cell marker Lysozyme and Cyptdin. Each symbol in bar graphs represents an ileum crypt (e,h), tissue sample (d), or organoid culture (g), isolated from one mouse. Data shown are representative (b,c,f) or combined (d,e,g,h) results from two independent reproducible experiments. Statistical significance is indicated using unpaired two-tailed t-test (e,h) or Two-way ANOVA with Tukey's multiple comparisons test (d,g). Data are presented as mean ± SD. Source data are provided as a Source Data file.

2a Gating strategy for the analysis of IFNγ⁺ CD4 or CD8 T cells in ileum lamina propria (LP)

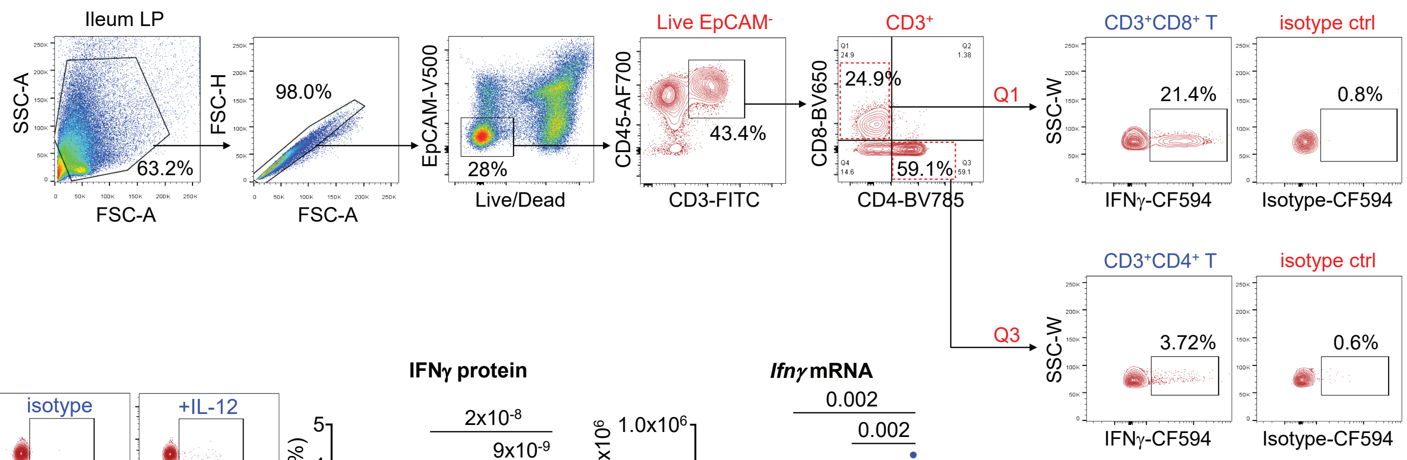

2b

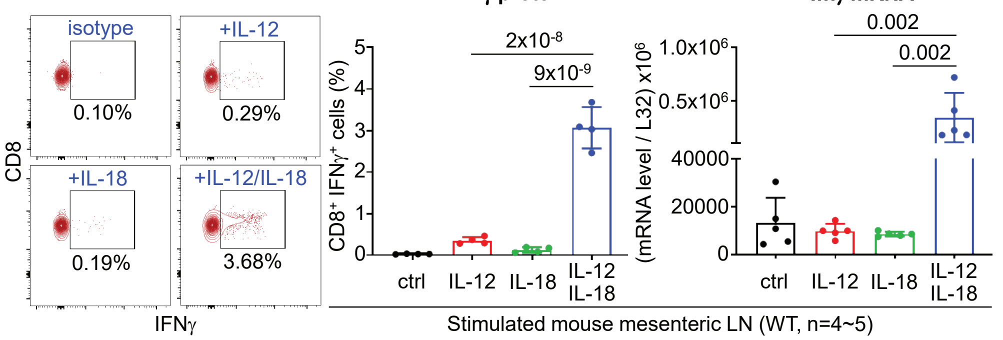

2c Gating strategy for the analysis of IFNγ-expressing cell populations in mesenteric lymph node (mLN)

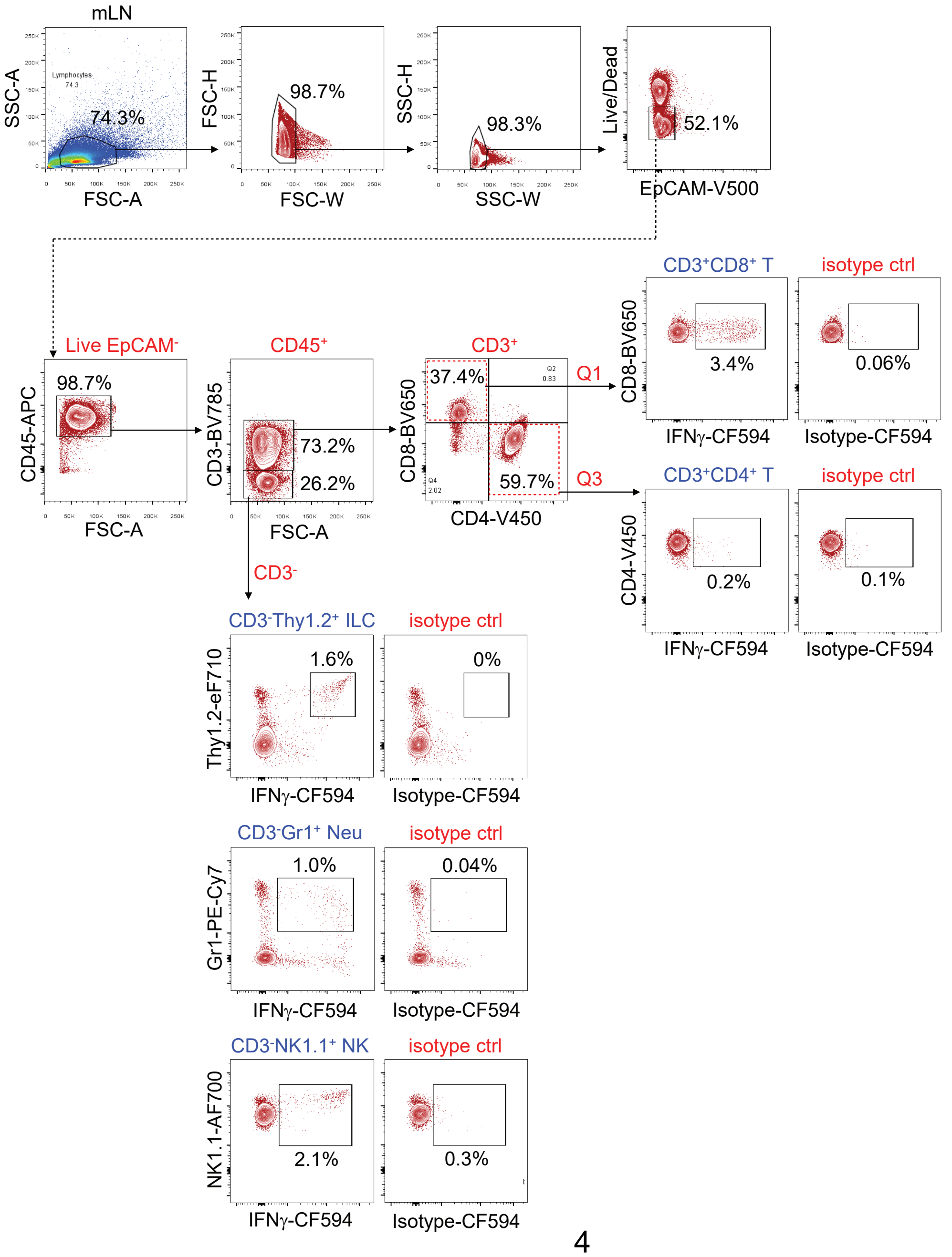

2d

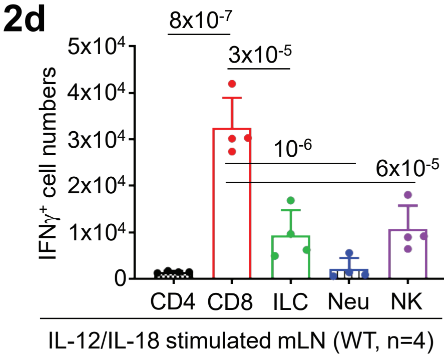
8x10-7
3x10-5
10-6
6x10-5
IFNγ+ cell numbers
5x104
4x104
3x104
2x104
1x104
0
CD4
CD8
ILC
Neu
NK
IL-12/IL-18 stimulated mLN (WT, n=4)

2e

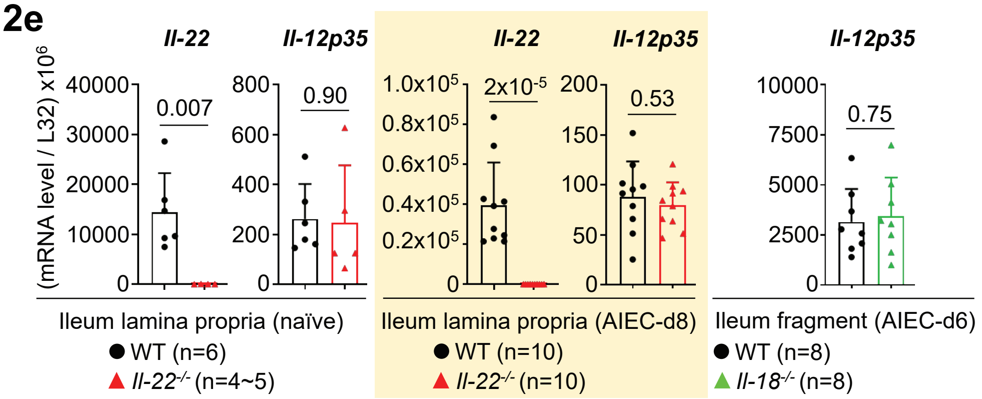
Il-22
Il-12p35
Il-22
Il-12p35
Il-12p35
(mRNA level / L32) x106
0.007
0.90
2x10-5
0.53
0.75
Ileum lamina propria (naïve)
WT (n=6)
Il-22-/- (n=4~5)
Ileum lamina propria (AIEC-d8)
WT (n=10)
Il-22-/- (n=10)
Ileum fragment (AIEC-d6)
WT (n=8)
Il-18-/- (n=8)

2f

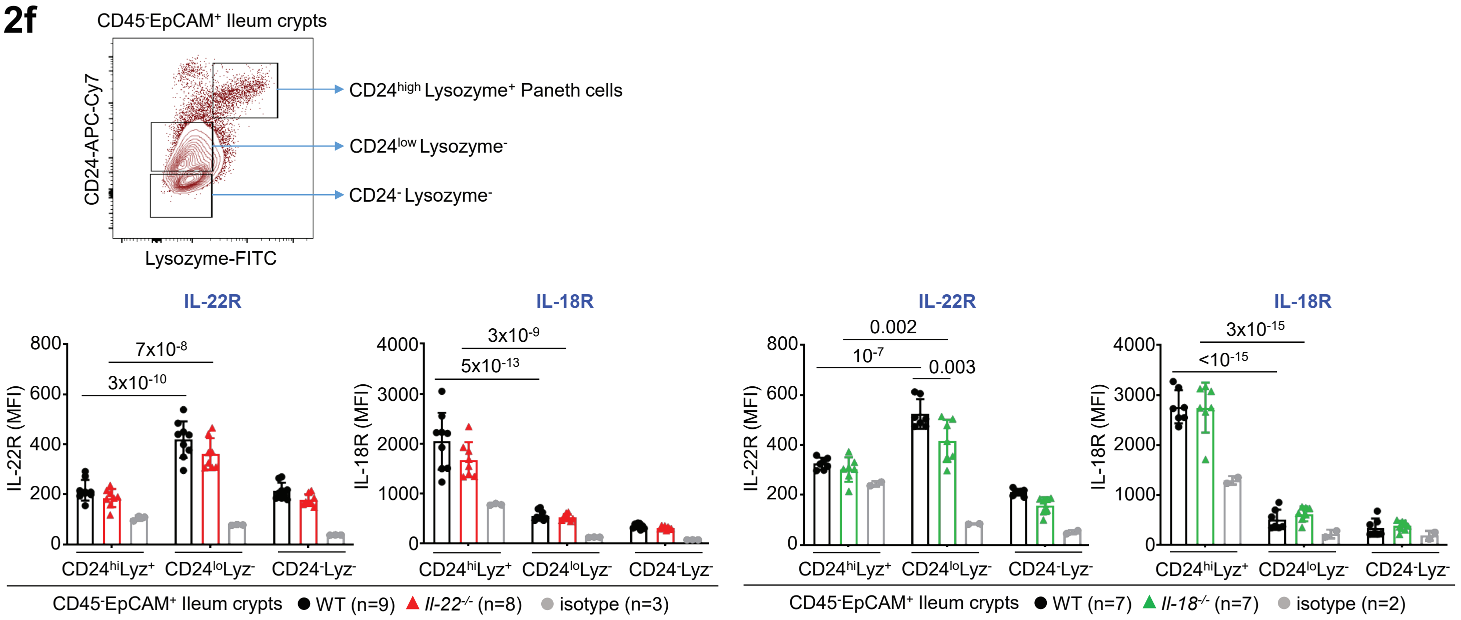
CD45-EpCAM+ Ileum crypts
CD24-APC-Cy7
Lysozyme-FITC
CD24high Lysozyme+ Paneth cells
CD24low Lysozyme-
CD24- Lysozyme-
IL-22R
IL-18R
IL-22R
IL-18R
7x10-8
3x10-10
3x10-9
5x10-13
0.002
10-7
0.003
3x10-15
<10-15
IL-22R (MFI)
IL-18R (MFI)
CD24hiLyz+
CD24loLyz-
CD24-Lyz-
CD45-EpCAM+ Ileum crypts
WT (n=9)
Il-22-/- (n=8)
isotype (n=3)
WT (n=7)
Il-18-/- (n=7)
isotype (n=2)

Supplementary Fig. 2. IL-12 and IL-18 promote IFNγ production in T cells.

(a) Gating strategy for flow cytometry analysis of PMA/Ionomycin (P/I)-stimulated ileum lamina propria (LP) cells for $IFN\gamma^{+}CD4^{+}$ or $IFN\gamma^{+}CD8^{+}$ T cells. The gating panels here correspond to FACS data panels in the manuscript figures 3b, 4i, and in the supplementary figure 2b. **(b)** Flow cytometry and quantitative real-time PCR analyses of IL-12/IL-18-stimulated mesenteric lymph node (mLN) cells for IFNγ in $CD8^{+}$ T cells. **(c)** Gating strategy for flow cytometry analysis of IL-12/IL-18-stimulated mLN cells for IFNγ. $CD3^{-}Thy1.2^{+}$ ILC (innate lymphoid cell), $CD3^{-}$ $Gr1^{+}$ Neu (neutrophil), and $CD3^{-}NK1.1^{+}$ NK cells within $CD45^{+}CD3^{-}$ cell sub-populations are indicated. Isotype antibodies are included as controls. The gating panels here correspond to FACS data panels in the supplementary figure 2d. **(d)** Flow cytometry analysis of IL-12/IL-18-stimulated mLN for intracellular IFNγ in the indicated cell populations. ILC ($CD3^{-}$ $Thy1.2^{+}$ innate lymphoid cells), Neu ($CD3^{-}$ $Gr1^{+}$ neutrophils), and NK ($CD3^{-}$ $NK1.1^{+}$ NK cells) are indicated. **(e)** Quantitative real-time PCR analysis of ileum lamina propria (LP) mononuclear cells or fragments from the indicated mice for *Il-22* or *Il-12p35* mRNA levels. **(f)** Flow cytometry analysis of ileum crypts from the indicated naïve littermate mice for IL-22R or IL-18R expression levels in the indicated epithelial subsets. Paneth cells are defined as $CD24^{+/high}$ $Lysozyme^{+}$ cells. Each symbol in bar graphs represents an ileum crypt (f) or intestinal tissue sample (b,d,e), isolated from one mouse. Data shown are representative (b,d) or combined (e,f) results from two independent reproducible experiments. Statistical significance is indicated using unpaired two-tailed t-test (e), One-way ANOVA with Sidak's multiple comparisons test (b,d), or Two-way ANOVA with Tukey's multiple comparisons test (f). Data are presented as mean ± SD. Source data are provided as a Source Data file.

3a Illustration of designed P1~P5 region within the mouse *Il-18* promoter for ChIP assay

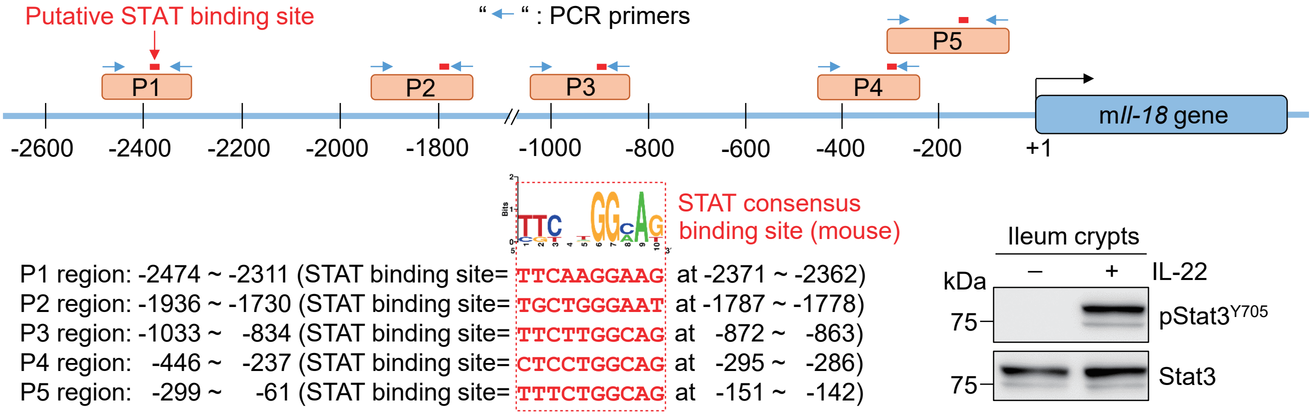

3b Promoter Co-occupancy Analysis by Chromatin Immunoprecipitation (ChIP)

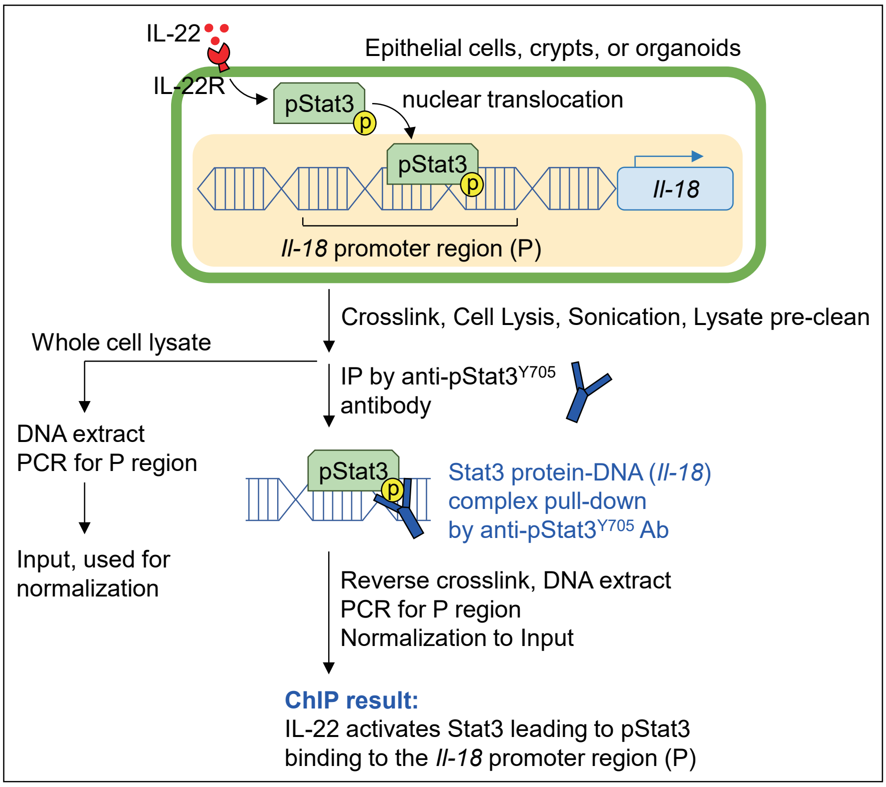

3c Illustration of designed P1~P3 region within the human *Il-18* promoter for ChIP assay

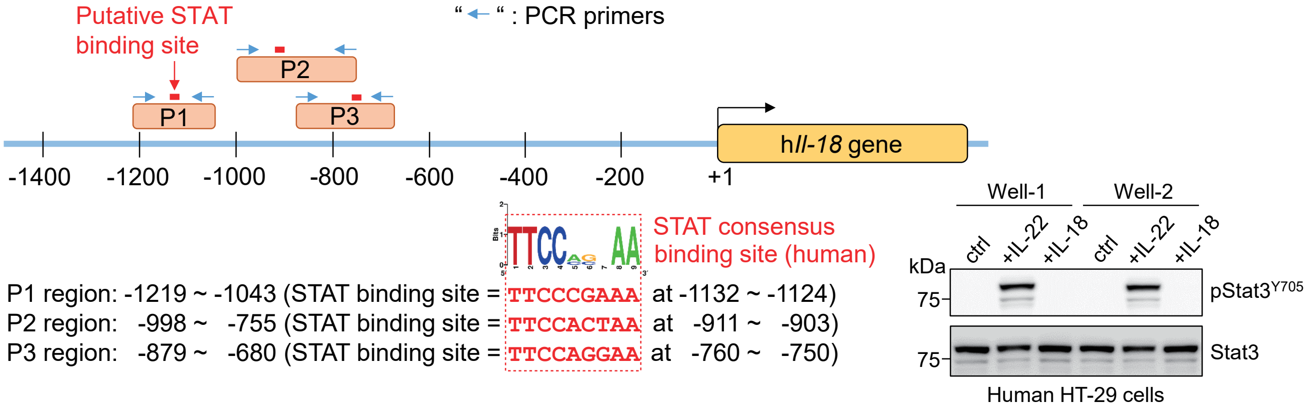

3d

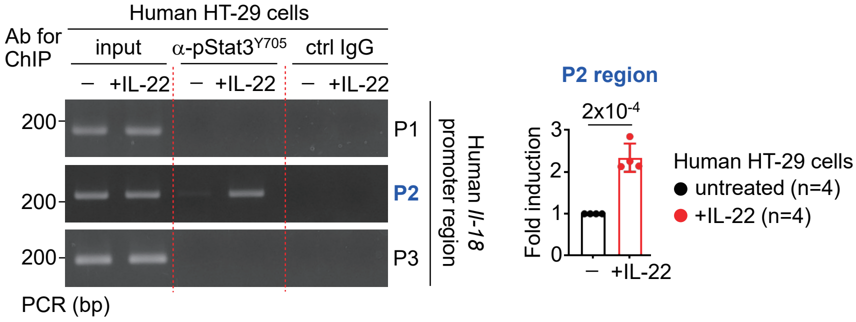

Supplementary Fig. 3. IL-22 induces the binding of phospho-Stat3 to the *Il-18* promoter.

(a) Graphic illustration of the designed P1~P5 region, each containing a STAT consensus binding site (indicated by a red square), within the mouse *Il-18* promoter for ChIP (chromatin immunoprecipitation) analysis. Pairs of PCR primers for each region are indicated by blue arrowheads. **(b)** Graphic illustration of promoter co-occupancy analysis by ChIP, using the *Il-18* promoter and pull-down of phospho-Stat3-bound *Il-18* DNA promoter region as an example. **(c)** Graphic illustration of the designed P1~P3 region, each containing a STAT consensus binding site (indicated by a red square), within the human *Il-18* promoter for ChIP analysis. Western blot analysis of IL-22 or IL-18-stimulated human colon epithelial HT-29 cells for Stat3 activation by a phospho-Stat3^{Y705} antibody. **(d)** ChIP and PCR analyses of IL-22-stimulated human HT-29 cells for the binding of phospho-Stat3^{Y705} to the human *Il-18* promoter regions (P1~P3). Fold induction is calculated based on PCR signal strength of phospho-Stat3^{Y705}-bound *Il-18* DNA promoter region (P2) before and after IL-22 stimulation. Each symbol in the bar graph (d) represents one well of HT-29 cell culture. Data shown are representative (a,c,d) results from two independent reproducible experiments. Statistical significance is indicated using unpaired two-tailed t-test (d). Data are presented as mean ± SD. Source data are provided as a Source Data file.

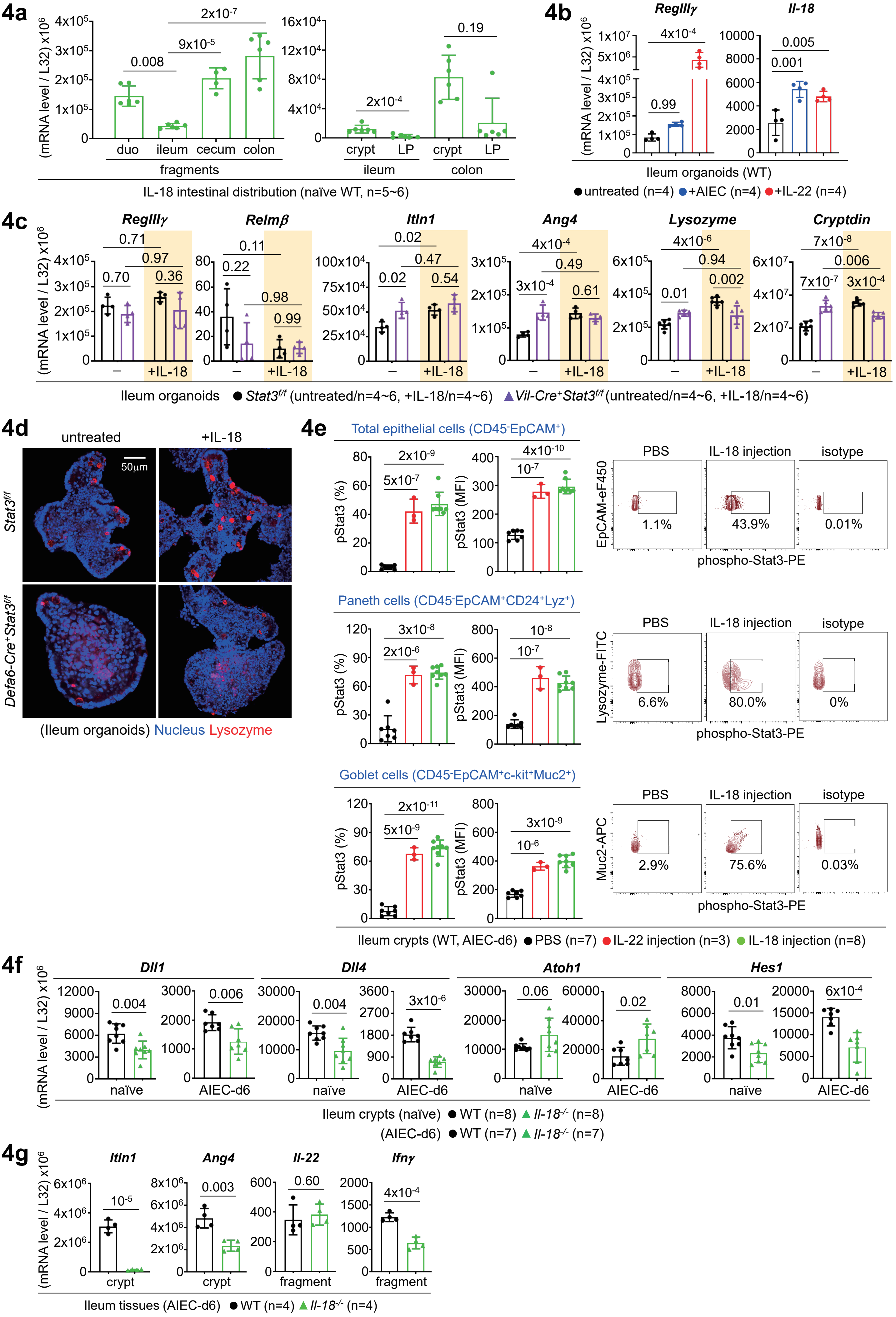
4a
IL-18 intestinal distribution (naïve WT, n=5~6)
fragments
duo ileum cecum colon
ileum colon
crypt LP crypt LP
(mRNA level / L32) x10⁶
4b
RegIIIγ
Il-18
Ileum organoids (WT)
untreated (n=4) +AIEC (n=4) +IL-22 (n=4)
4c
RegIIIγ Relmβ Itln1 Ang4 Lysozyme Cryptdin
+IL-18
Ileum organoids Stat3f/f (untreated/n=4~6, +IL-18/n=4~6) Vil-Cre+Stat3f/f (untreated/n=4~6, +IL-18/n=4~6)
4d
untreated +IL-18
50μm
Stat3f/f
Defa6-Cre+Stat3f/f
(Ileum organoids) Nucleus Lysozyme
4e
Total epithelial cells (CD45-EpCAM+)
Paneth cells (CD45-EpCAM+CD24+Lyz+)
Goblet cells (CD45-EpCAM+c-kit+Muc2+)
pStat3 (%)
pStat3 (MFI)
PBS IL-18 injection isotype
EpCAM-eF450
1.1% 43.9% 0.01%
Lysozyme-FITC
6.6% 80.0% 0%
Muc2-APC
2.9% 75.6% 0.03%
phospho-Stat3-PE
Ileum crypts (WT, AIEC-d6) PBS (n=7) IL-22 injection (n=3) IL-18 injection (n=8)
4f
Dll1 Dll4 Atoh1 Hes1
naïve AIEC-d6
Ileum crypts (naïve) WT (n=8) Il-18-/- (n=8)
(AIEC-d6) WT (n=7) Il-18-/- (n=7)
4g
Itln1 Ang4 Il-22 Ifnγ
crypt crypt fragment fragment
Ileum tissues (AIEC-d6) WT (n=4) Il-18-/- (n=4)

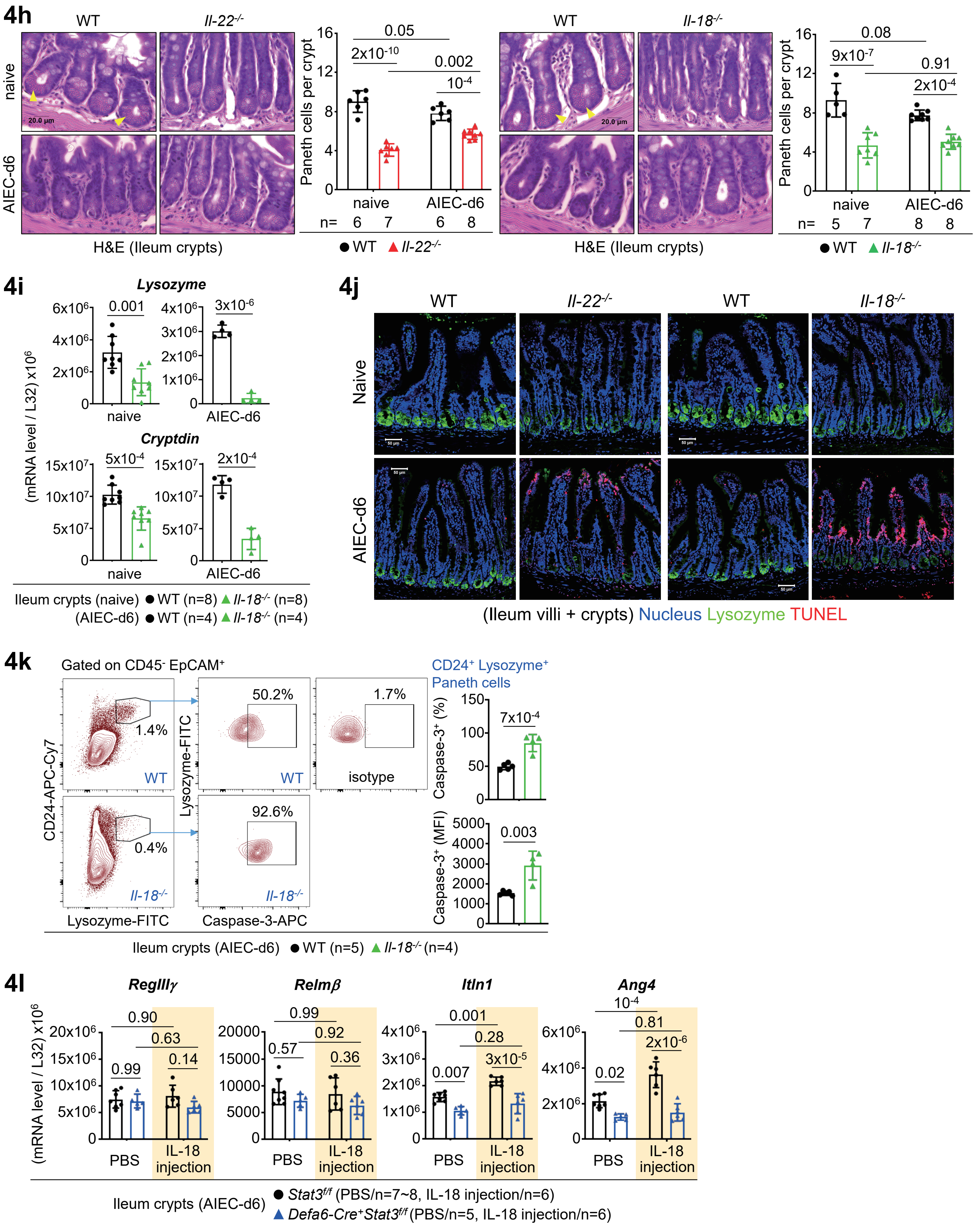
4h
WT
Il-22-/-
naive
AIEC-d6
20.0 µm
Paneth cells per crypt
0.05
2x10-10
0.002
10-4
naive
AIEC-d6
n= 6 7 6 8
H&E (Ileum crypts)
● WT ▲ Il-22-/-
WT
Il-18-/-
0.08
9x10-7
0.91
2x10-4
naive
AIEC-d6
n= 5 7 8 8
H&E (Ileum crypts)
● WT ▲ Il-18-/-
4i
Lysozyme
0.001
3x10-6
naive
AIEC-d6
Cryptdin
5x10-4
2x10-4
(mRNA level / L32) x10^6
Ileum crypts (naive) ● WT (n=8) ▲ Il-18-/- (n=8)
(AIEC-d6) ● WT (n=4) ▲ Il-18-/- (n=4)
4j
WT
Il-22-/-
WT
Il-18-/-
Naive
AIEC-d6
(Ileum villi + crypts) Nucleus Lysozyme TUNEL
4k
Gated on CD45- EpCAM+
CD24-APC-Cy7
1.4%
WT
0.4%
Il-18-/-
Lysozyme-FITC
Lysozyme-FITC
50.2%
WT
1.7%
isotype
92.6%
Il-18-/-
Caspase-3-APC
CD24+ Lysozyme+ Paneth cells
Caspase-3+ (%)
7x10-4
Caspase-3+ (MFI)
0.003
Ileum crypts (AIEC-d6) ● WT (n=5) ▲ Il-18-/- (n=4)
4l
RegIIIγ
0.90
0.63
0.99
0.14
Relmβ
0.99
0.57
0.92
0.36
Itln1
0.001
0.28
0.007
3x10-5
Ang4
10-4
0.81
2x10-6
0.02
(mRNA level / L32) x10^6
PBS
IL-18 injection
Ileum crypts (AIEC-d6)
● Stat3f/f (PBS/n=7~8, IL-18 injection/n=6)
▲ Defa6-Cre+Stat3f/f (PBS/n=5, IL-18 injection/n=6)

Supplementary Fig. 4. IL-18 is a bona fide regulator of Paneth cells.

(a) Quantitative real-time PCR analysis of fragments, crypts, or lamina propria (LP) cells, isolated from the indicated intestinal tissues of naïve mice, for *Il-18* mRNA levels. **(b)** Quantitative real-time PCR analysis of AIEC or IL-22-stimulated ileum organoids for anti-microbial RegIIIγ and IL-18. **(c)** Quantitative real-time PCR analysis of IL-18-stimulated ileum organoids, derived from the indicated mice, for anti-microbial peptides. **(d)** Immunofluorescence analysis of IL-18-stimulated ileum organoids, derived from the indicated mice, for Lysozyme$^+$ Paneth cells at day-5 of culture. **(e)** Flow cytometry analysis of ileum crypts, derived from PBS, IL-22 or IL-18-injected wild-type mice at day-6 (d6) post AIEC infection, for phospho-Stat3 (% and MFI) in the indicated epithelial subsets. **(f)** Quantitative real-time PCR analysis of ileum crypts for Notch ligand (Dll1, Dll4), transcription factor for secretory precursor (Atoh1), and Notch target gene (Hes1) in the indicated uninfected or AIEC-infected mice. **(g)** Quantitative real-time PCR analysis of ileum crypts or fragments for anti-microbial peptides or cytokines in the indicated AIEC-infected mice. **(h)** H&E staining analysis of ileum crypts from the indicated uninfected or AIEC-infected littermate mice for granule-containing Paneth cells (indicated by yellow arrowheads). Quantification of Paneth cells per crypt is indicated. **(i)** Quantitative real-time PCR analysis of ileum crypts from the indicated mice for Paneth cell marker Lysozyme and Cryptdin. **(j)** Immunofluorescence analysis of ileum tissues, derived from the indicated uninfected or AIEC-infected littermate mice at day-6 (d6), for Lysozyme$^+$ Paneth cells and/or TUNEL$^+$ apoptotic cells. **(k)** Flow cytometry analysis of ileum crypts, derived from the indicated mice at d6 post AIEC infection, for active Caspase-3$^+$ apoptotic cells in the gated Lysozyme$^+$ Paneth cells. **(l)** Quantitative real-time PCR analysis of ileum crypts from the indicated PBS or IL-18-injected mice at d6 post AIEC infection for anti-microbial peptides. Each symbol in bar graphs represents an ileum crypt sample (a,e,f,g,i,k,l), tissue sample (a,g,h), or ileum organoids (b,c), derived from one mouse. Data shown are representative (b,d,g,j,k) or combined (a,c,e,f,h,i,l) results from two independent reproducible experiments. Statistical significance is indicated using unpaired two-tailed t-test (a,f,g,i,k), One-way ANOVA with Sidak's multiple comparisons test (a,b,e), or Two-way ANOVA with Tukey's multiple comparisons test (c,h,l). Data are presented as mean ± SD. Source data are provided as a Source Data file.

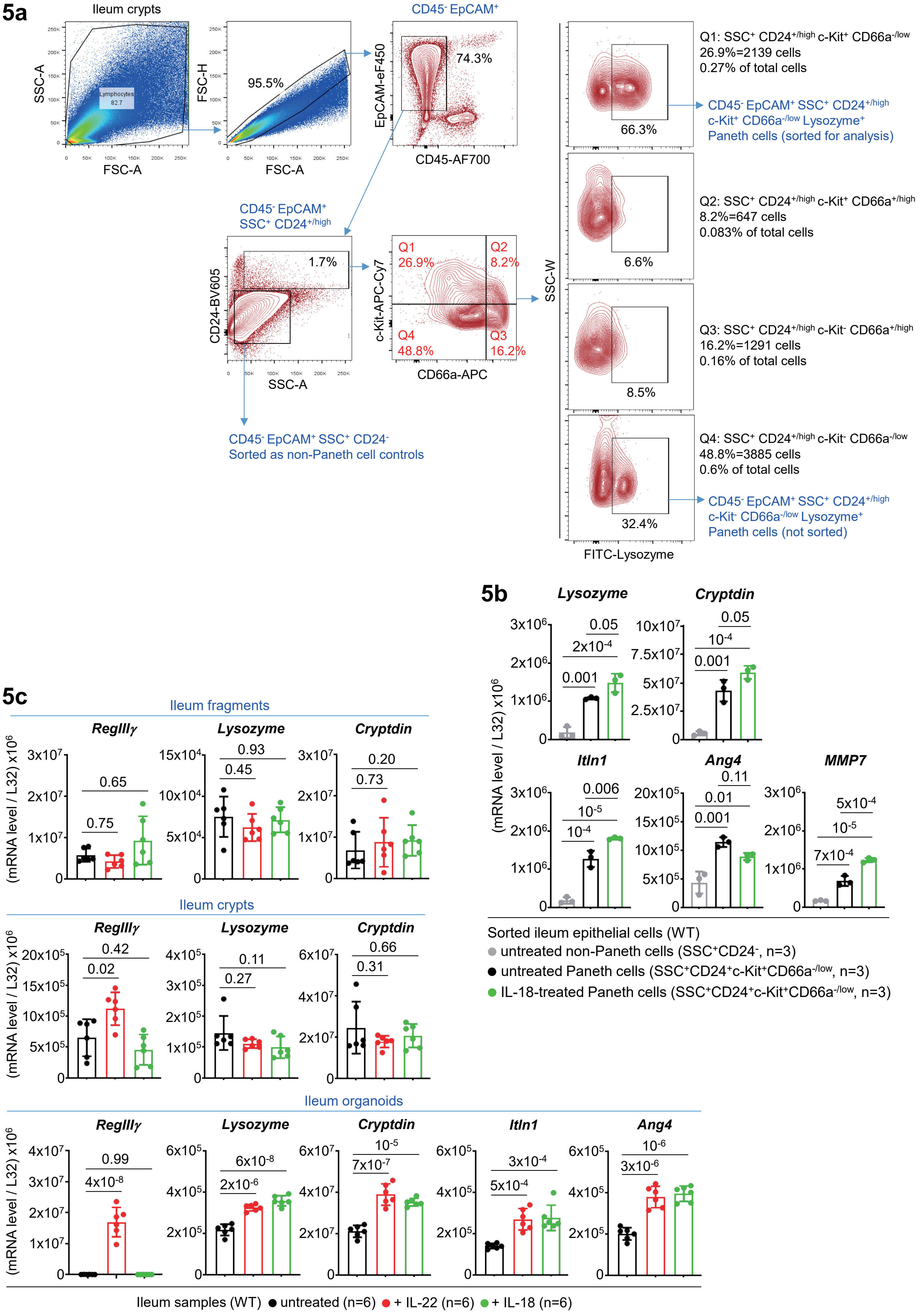

5a
Ileum crypts
SSC-A
FSC-A
Lymphocytes 82.7
FSC-H
95.5%
CD45- EpCAM+
EpCAM-eF450
74.3%
CD45-AF700
CD45- EpCAM+ SSC+ CD24+/high
CD24-BV605
1.7%
SSC-A
c-Kit-APC-Cy7
Q1 26.9%
Q2 8.2%
Q4 48.8%
Q3 16.2%
CD66a-APC
CD45- EpCAM+ SSC+ CD24-
Sorted as non-Paneth cell controls
SSC-W
66.3%
6.6%
8.5%
32.4%
FITC-Lysozyme
Q1: SSC+ CD24+/high c-Kit+ CD66a-/low
26.9%=2139 cells
0.27% of total cells
CD45- EpCAM+ SSC+ CD24+/high c-Kit+ CD66a-/low Lysozyme+
Paneth cells (sorted for analysis)
Q2: SSC+ CD24+/high c-Kit+ CD66a+/high
8.2%=647 cells
0.083% of total cells
Q3: SSC+ CD24+/high c-Kit- CD66a+/high
16.2%=1291 cells
0.16% of total cells
Q4: SSC+ CD24+/high c-Kit- CD66a-/low
48.8%=3885 cells
0.6% of total cells
CD45- EpCAM+ SSC+ CD24+/high c-Kit- CD66a-/low Lysozyme+
Paneth cells (not sorted)
5b
Lysozyme
Cryptdin
Itln1
Ang4
MMP7
(mRNA level / L32) x10^6
Sorted ileum epithelial cells (WT)
untreated non-Paneth cells (SSC+CD24-, n=3)
untreated Paneth cells (SSC+CD24+c-Kit+CD66a-/low, n=3)
IL-18-treated Paneth cells (SSC+CD24+c-Kit+CD66a-/low, n=3)
5c
Ileum fragments
Ileum crypts
Ileum organoids
RegIIIγ
Lysozyme
Cryptdin
Itln1
Ang4
(mRNA level / L32) x10^6
Ileum samples (WT) untreated (n=6) + IL-22 (n=6) + IL-18 (n=6)

Supplementary Fig. 5. IL-18 directly regulates Paneth cells.

(a) Gating strategy for sorting $CD45^-$ $EpCAM^+$ SSC^+ $CD24^{+/high}$ c-Kit^+ $CD66a^{-/low}$ Paneth cells from ileum crypts by flow cytometry. The indicated gated Paneth cells (Q1 and Q4 by surface staining of c-Kit and CD66a) are tested positive for intracellular Lysozyme. Only Q1 Paneth cells were sorted for the culture experiment. $CD45^-$ $EpCAM^+$ SSC^+ $CD24^-$ non-Paneth cells are sorted and used as a negative control for the analysis. The gating panels here correspond to FACS data panels in the supplementary figure 5b. **(b)** Quantitative real-time PCR analysis of IL-18-stimulated sorted Paneth cells, for Paneth cell-specific anti-microbial peptides (AMP) (Lysozyme, Cryptdin) and Paneth cell-related AMP (Itln1, Ang4, MMP7). **(c)** Quantitative real-time PCR analysis of IL-22 or IL-18-stimulated ileum fragments (upper panels), fresh ileum crypts (middle panels), or ileum organoids (lower panels), derived from wild-type mice, for anti-microbial peptides. Each symbol in bar graph (b) represents an epithelial cell subset sorted from combined ileum crypts of three mice. Each symbol in bar graph (c) represents an ileum sample derived from one mouse. Data shown are combined (b,c) results from two (c) or three (b) independent reproducible experiments. Statistical significance is indicated with One-way ANOVA with Tukey's multiple comparisons test (b), or with Sidak's multiple comparisons test (c). Data are presented as mean ± SD. Source data are provided as a Source Data file.

6a

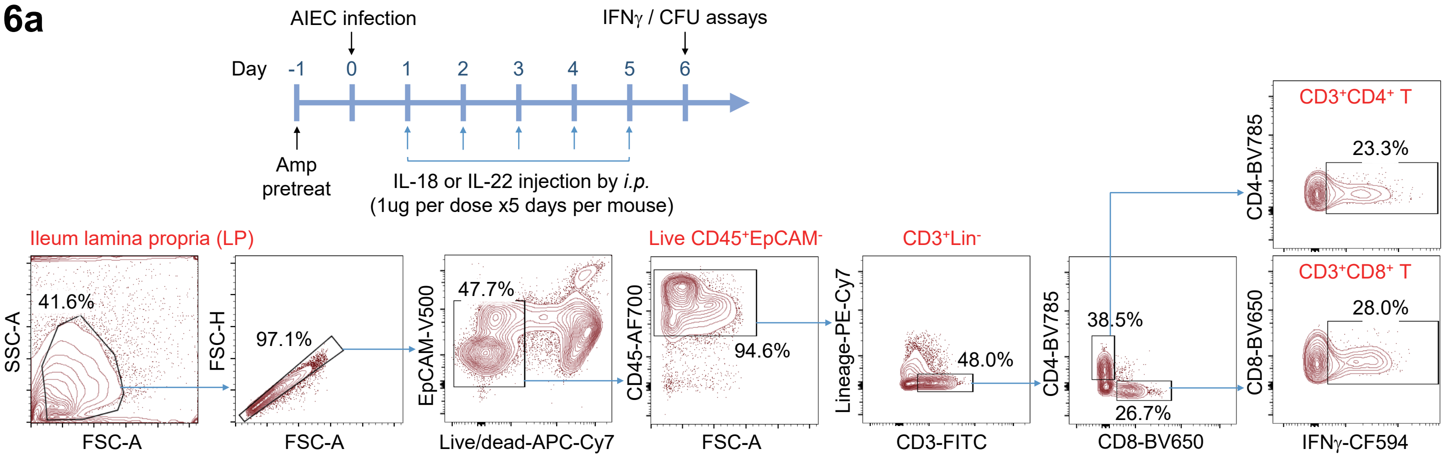
AIEC infection
IFNγ / CFU assays
Day -1 0 1 2 3 4 5 6
Amp pretreat
IL-18 or IL-22 injection by i.p.
(1ug per dose x5 days per mouse)
Ileum lamina propria (LP)
Live CD45+EpCAM-
CD3+Lin-
CD3+CD4+ T
CD3+CD8+ T
SSC-A
FSC-A
41.6%
FSC-H
97.1%
EpCAM-V500
Live/dead-APC-Cy7
47.7%
CD45-AF700
94.6%
Lineage-PE-Cy7
CD3-FITC
48.0%
CD4-BV785
CD8-BV650
38.5%
26.7%
IFNγ-CF594
23.3%
28.0%

6b

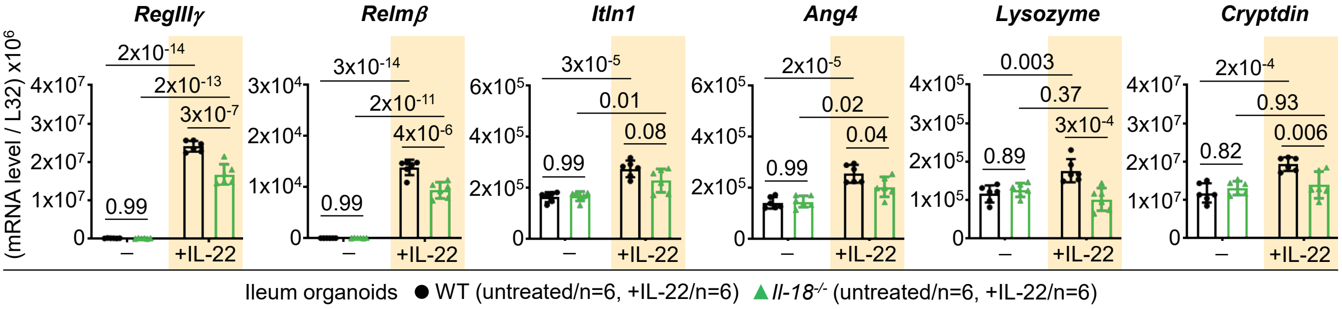
RegIIIγ
Relmβ
Itln1
Ang4
Lysozyme
Cryptdin
(mRNA level / L32) x10^6
–
+IL-22
Ileum organoids ● WT (untreated/n=6, +IL-22/n=6) ▲ Il-18-/- (untreated/n=6, +IL-22/n=6)

Supplementary Fig. 6. IL-22 links IL-18 for IFNγ and Paneth cell responses.

(a) The protocol for in vivo rescue experiment and gating strategy for flow cytometry analysis of ileum lamina propria (LP) cells for IFNγ^{+}CD4^{+} or IFNγ^{+}CD8^{+} T cells. Mice were given 40 mg amplicillin by oral gavage one day before adherent invasive *E. coli* (AIEC) oral infection. The gating panels here correspond to FACS data panels in the manuscript figures 5b and 5e. **(b)** Quantitative real-time PCR analysis of IL-22-stimulated ileum organoids, derived from the indicated mice, for anti-microbial peptides. Each symbol in the bar graph (b) represents an ileum organoid sample derived from one mouse. Data shown are combined (b) results from two independent reproducible experiments. Statistical significance is indicated with Two-way ANOVA with Tukey's multiple comparisons test (b). Data are presented as mean ± SD. Source data are provided as a Source Data file.

7a

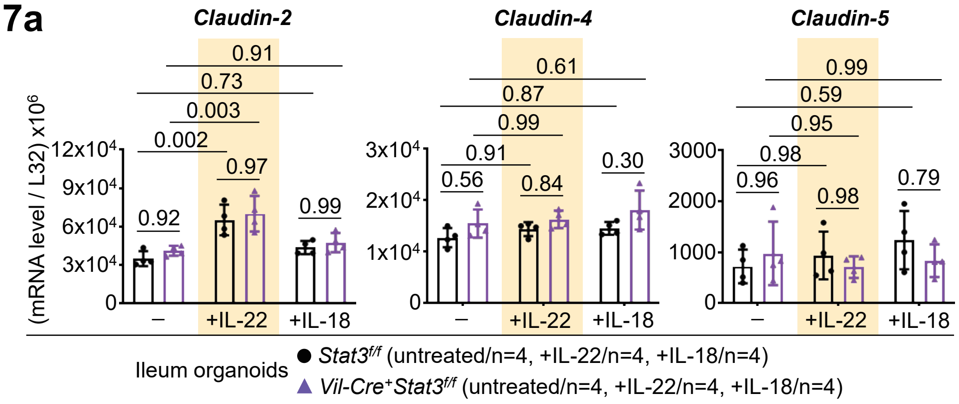
Claudin-2
Claudin-4
Claudin-5
(mRNA level / L32) x10^6
12x10^4
9x10^4
6x10^4
3x10^4
0
3x10^4
2x10^4
1x10^4
3000
2000
1000
0.91
0.73
0.003
0.002
0.97
0.92
0.99
0.61
0.87
0.99
0.91
0.56
0.84
0.30
0.99
0.59
0.95
0.98
0.96
0.98
0.79
–
+IL-22
+IL-18
Ileum organoids
● Stat3^f/f (untreated/n=4, +IL-22/n=4, +IL-18/n=4)
▲ Vil-Cre^+Stat3^f/f (untreated/n=4, +IL-22/n=4, +IL-18/n=4)

7b

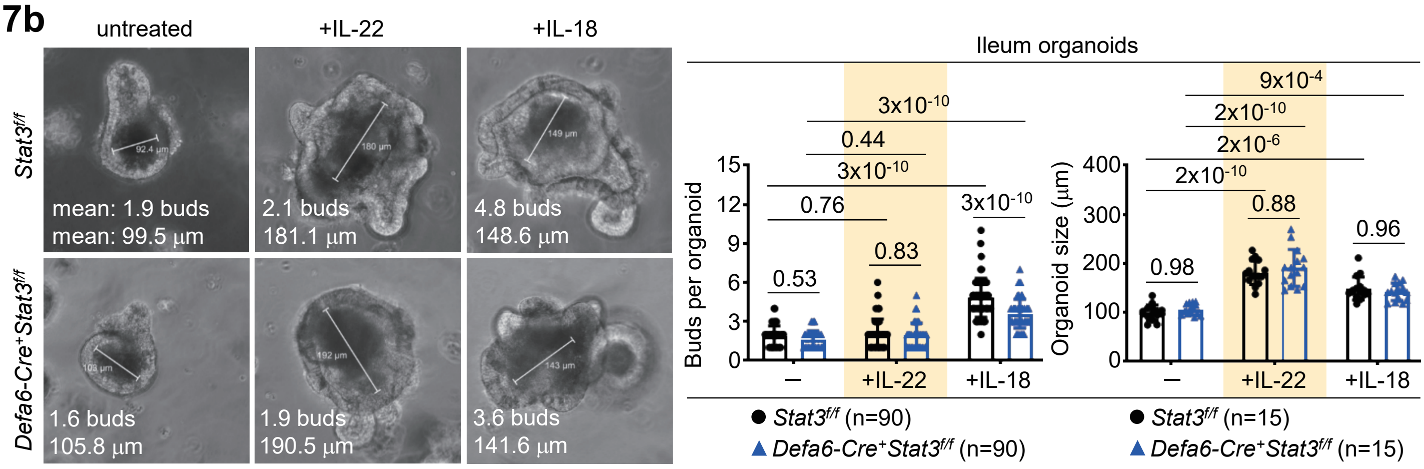
untreated
+IL-22
+IL-18
Stat3^f/f
Defa6-Cre^+Stat3^f/f
mean: 1.9 buds
mean: 99.5 µm
2.1 buds
181.1 µm
4.8 buds
148.6 µm
1.6 buds
105.8 µm
1.9 buds
190.5 µm
3.6 buds
141.6 µm
Ileum organoids
Buds per organoid
15
12
9
6
3
0
3x10^-10
0.44
3x10^-10
0.76
3x10^-10
0.83
0.53
Organoid size (µm)
400
300
200
100
9x10^-4
2x10^-10
2x10^-6
2x10^-10
0.88
0.96
0.98
–
+IL-22
+IL-18
● Stat3^f/f (n=90)
▲ Defa6-Cre^+Stat3^f/f (n=90)
● Stat3^f/f (n=15)
▲ Defa6-Cre^+Stat3^f/f (n=15)

Supplementary Fig. 7. Differential role of IL-22 and IL-18 in organoid culture.

(a) Quantitative real-time PCR analysis of IL-22 or IL-18-stimulated ileum organoids, derived from the indicated mice, for tight junction protein gene *Claudin-2/-4/-5.* Each symbol in bar graphs represents an organoid culture derived from one mouse. **(b)** Quantification of the number of buds per organoid and size in IL-22 or IL-18-stimulated ileum organoids, derived from the indicated mice. "n" indicates the number of images taken from organoids derived from three mice in each group. Data shown are representative (a) or combined (b) results from two independent reproducible experiments. Statistical significance is indicated using Two-way ANOVA with Tukey's multiple comparisons test (a,b). Data are presented as mean ± SD. Source data are provided as a Source Data file.

8a

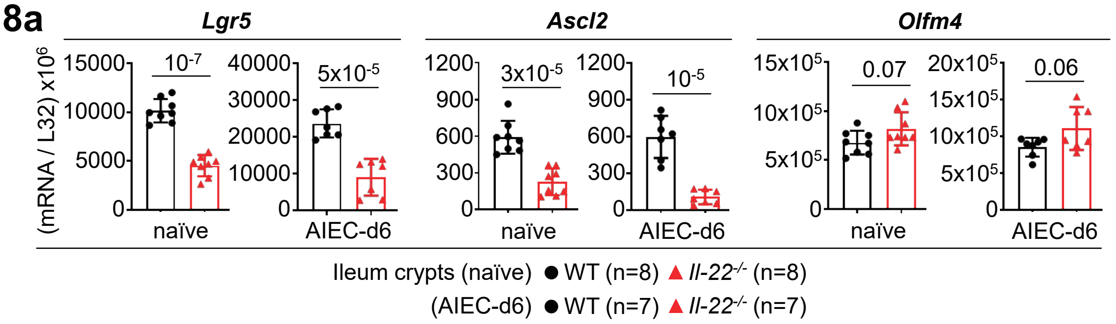

8b

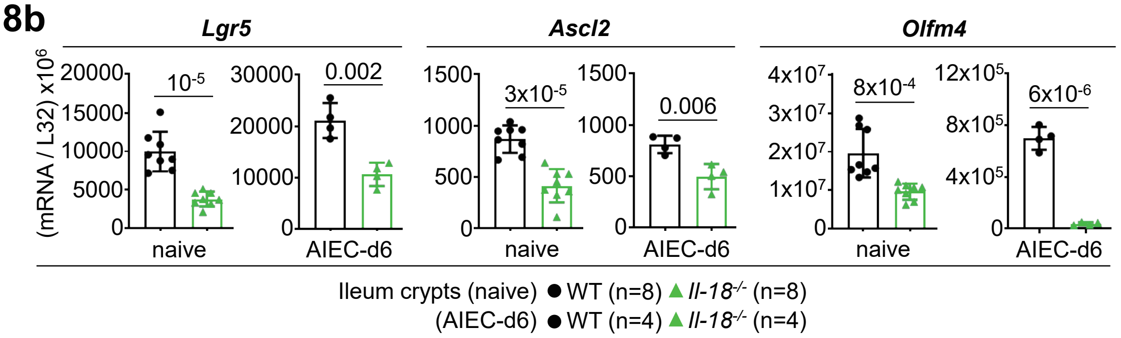

8c Illustration of designed P1~P6 region within the mouse *Lgr5* promoter for ChIP assay

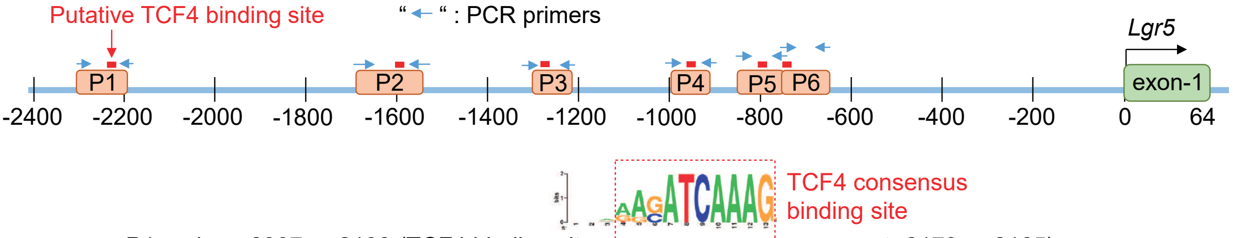

P1 region: -2307 ~ -2188 (TCF4 binding site= **GACATCAAAG**GGCGT at -2179 ~ -2165)
P2 region: -1680 ~ -1554 (TCF4 binding site= **CTTATCAAGG**AGAAA at -1599 ~ -1585)
P3 region: -1296 ~ -1238 (TCF4 binding site= **GTTGGCAAAG**TAGGC at -1281 ~ -1267)
P4 region: -989 ~ -927 (TCF4 binding site= **TGGTTTTAAG**ATCCC at -960 ~ -946)
P5 region: -861 ~ -748 (TCF4 binding site= **ACTATCAAAG**CCTCA at -804 ~ -790)
P6 region: -767 ~ -650 (TCF4 binding site= **AGGATCAAAA**AGGCC at -755 ~ -741)

Supplementary Fig. 8. IL-22 and IL-18 regulate Lgr5$^+$ stem cells during AIEC infection.

(a,b) Quantitative real-time PCR analysis of ileum crypts, isolated from the indicated uninfected or AIEC-infected mice at day-6 (d6) post infection, for stem cell gene Lgr5, Ascl2, and Olfm4. **(c)** Graphic illustration of the designed P1~P6 region, each containing a TCF4 consensus binding site (indicated by a red square) within the mouse *Lgr5* promoter, for ChIP (chromatin immunoprecipitation) analysis. Pairs of PCR primers for each region are indicated by blue arrowheads. Each symbol in bar graphs represents an ileum crypt sample (a,b) derived from one mouse. Data shown are combined (a,b) results from two independent reproducible experiments. Statistical significance is indicated using unpaired two-tailed t-test (a,b). Data are presented as mean ± SD. Source data are provided as a Source Data file.

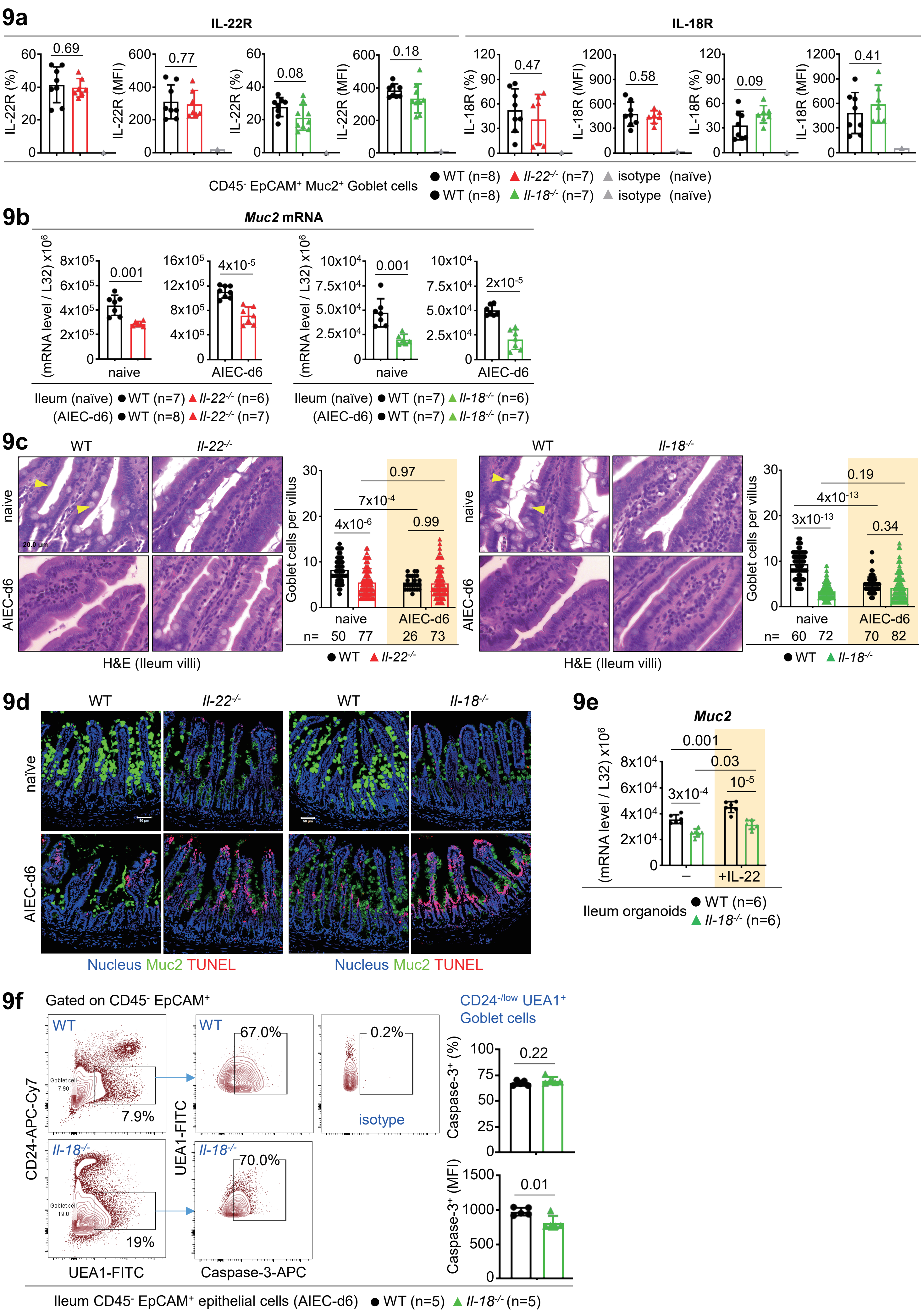

9a
IL-22R
IL-18R
IL-22R (%)
IL-22R (MFI)
IL-18R (%)
IL-18R (MFI)
0.69
0.77
0.08
0.18
0.47
0.58
0.09
0.41
CD45- EpCAM+ Muc2+ Goblet cells
WT (n=8) Il-22-/- (n=7) isotype (naïve)
WT (n=8) Il-18-/- (n=7) isotype (naïve)
9b
Muc2 mRNA
(mRNA level / L32) x10^6
0.001
4x10-5
2x10-5
naive
AIEC-d6
Ileum (naïve) WT (n=7) Il-22-/- (n=6)
(AIEC-d6) WT (n=8) Il-22-/- (n=7)
Ileum (naïve) WT (n=7) Il-18-/- (n=6)
(AIEC-d6) WT (n=7) Il-18-/- (n=7)
9c
WT
Il-22-/-
Il-18-/-
naive
AIEC-d6
20.0 µm
Goblet cells per villus
0.97
7x10-4
4x10-6
0.99
0.19
4x10-13
3x10-13
0.34
n= 50 77 26 73
n= 60 72 70 82
WT Il-22-/-
WT Il-18-/-
H&E (Ileum villi)
9d
Nucleus Muc2 TUNEL
9e
Muc2
0.001
0.03
3x10-4
10-5
–
+IL-22
Ileum organoids
WT (n=6)
Il-18-/- (n=6)
9f
Gated on CD45- EpCAM+
CD24-/low UEA1+ Goblet cells
WT
Il-18-/-
7.9%
19%
67.0%
0.2%
70.0%
isotype
CD24-APC-Cy7
UEA1-FITC
Caspase-3-APC
Caspase-3+ (%)
Caspase-3+ (MFI)
0.22
0.01
Ileum CD45- EpCAM+ epithelial cells (AIEC-d6) WT (n=5) Il-18-/- (n=5)

Supplementary Fig. 9. IL-22 and IL-18 regulate Goblet cells during AIEC infection.

(a) Flow cytometry analysis of ileum epithelial cells, isolated from the indicated naïve mice, for IL-22R and IL-18R expression in the gated Muc2$^+$ Goblet cells. **(b)** Quantitative real-time PCR analysis of ileum epithelial cells, isolated from the indicated uninfected or AIEC-infected mice at day-6 (d6) post infection, for *Muc2* mRNA. **(c)** H&E staining analysis of ileum villi from the indicated uninfected or AIEC-infected littermate mice (n=3 in each group) at d6 post infection for Goblet cells, which are indicated by yellow arrowheads. Quantification of Goblet cells per villus is shown. **(d)** Immunofluorescence analysis of ileum tissues, derived from the indicated uninfected or AIEC-infected littermate mice (n=3 in each group) at d6 post infection, for Muc2$^+$ Goblet cells and TUNEL$^+$ apoptotic cells. **(e)** Quantitative real-time PCR analysis of IL-22-stimulated ileum organoids, derived from the indicated mice, for Goblet cell gene *Muc2*. **(f)** Flow cytometry analysis of ileum epithelial cells, isolated from the indicated AIEC-infected mice at d6 post infection, for Caspase-3$^+$ apoptotic cells in the gated UEA1$^+$ Goblet cells. Each symbol in bar graphs represents an ileum epithelial sample (a,b,f), or ileum organoid (e), derived from one mouse. Data shown are representative (c,d,f) or combined (a,b,e) results from two independent reproducible experiments. Statistical significance is indicated using unpaired two-tailed t-test (a,b,f), or Two-way ANOVA with Tukey's multiple comparisons test (c,e). Data are presented as mean ± SD. Source data are provided as a Source Data file.

Supplementary Table 1. List of primers for quantitative real-time PCR

Gene Name	Froward (5' to 3')	Reverse (5' to 3')
L32	GAAACTGGCGGAAACCCA	GGATCTGGCCCTTGAACCTT
Lgr5	CGAGCCTTACAGAGCCTGATACC	TTGCCGTCGTCTTTATTCCATTGG
Ascl2	CCTCTCTCGGACCCTCTCTCAG	CAGTCAAGGTGTGCTTCCATGC
Olfm4	GCCACTTTCCAATTTCAC	GAGCCTCTTCTCATACAC
Lyz1	GCCAAGGTCTAACAATCGTTGTGAGTTG	CAGTCAGCCAGCTTGACACCACG
Cryptdin	AGGAGCAGCCAGGAGAAG	ATGTTCAGCGACAGCAGAG
Mmp7	CAGACTTACCTCGGATCGTAGTGG	GTTCACTCCTGCGTCCTCACC
RegIIIγ	TCAGGTGCAAGGTGAAGTTG	GGCCACTGTTACCACTGCTT
Relmβ	CCATTTCCTGAGCTTTCTGG	AGCACATCCAGTGACAACCA
Itln1	TCCAGTCAGCAAGGCAACAGAG	CAGGTTCTCAGCCTGGATGTCA
Ang4	GGCACCAAGAAAAACATCAGGGC	GTGCGTACAAGTGGTGATCTGG
Il-18	TGAAGTAAGAGGACTGGCTGTGAC	ATCTTGTTGTGTCCTGGAACACG
Il-22	TCGCCTTGATCTCTCCACTC	GCTCAGCTCCTGTCACATCA
Il-12p35	GCTTCTCCCACAGGAGGTTT	CTAGACAAGGGCATGCTGGT
Ifnγ	CAGCAACAGCAAGGCGAAAAAGG	TTTCCGCTTCCTGAGGCTGGAT
Cldn2	TGCGACACACAGCACAGGCATCAC	TCAGGAACCAGCGGCGAGTAGAA
Cldn4	GGCGTAATGGCAAGAGTAGC	CTCGGAGTGGATGTCCTCAT
Cldn5	GTCACGATGTTGTGGTCCAG	AAATTCTGGGTCTGGTGCTG

Supplementary Table 2. ChIP primers for *Il-18* promoter region in HT-29

Stat3 binding site region	Froward (5' to 3')	Reverse (5' to 3')
P1	CTGATAGTCACCACACCAAGCAC	CTAGAGTCAGGGAACAAAATGCC
P2	AACAAAATGCCACCTTGCTAAT	TGGAATGATAGCAAAGACTGACC
P3	ACACTCTGCTCTTCAAACGT	GGGAGAGGGATACCAAAAT

Supplementary Table 3. ChIP primers for *Il-18* promoter region in mouse ileum crypt

Stat3 binding site region	Froward (5' to 3')	Reverse (5' to 3')
P1	ACACAATACATAAGCCTAAAATGTCT	GTTCCTCTTCTTGGGTTGGGA
P2	TGAACAGTCCAACTAGATACGCA	GCTCAGTGGTTAAGGACGCT
P3	CCCCAGGCTCAAAATTCTGC	GTTTCTCTTTCCAGACTTCAACTGA
P4	GCATATGCAACTCAACCTTCAAGT	TAGGTGCTAGGAGTGGGGAG
P5	ACCTCTCCTGGCAGTATCCT	ACCCTGTGGTTTTTGTTCTGT

Supplementary Table 4. ChIP primers for *Lgr5* promoter region in mouse ileum organoid and CMT93 cells

TCF4 binding site region	Froward (5' to 3')	Reverse (5' to 3')
P1	GCAACGCTATGCTCCTTTGG	TTGAGGGAAGGGTCTAGGCA
P5	GAATTGGGAGGACCCTGGAG	TTGATCCTTCCATCAGGGCG

Supplementary Methods

Cell culture

Mouse epithelial adherent rectal polyploid carcinoma cell line CMT93 (ATCC #CCL-223), human epithelial adherent colorectal adenocarcinoma cell line HT-29 (ATCC #HTB-38™), and human epithelial adherent embryonic kidney cell line HEK293 (ATCC #CRL-1573™) were purchased from American Type Culture Collection (ATCC) and used for in vitro studies. The cells were maintained as a monolayer in Dulbecco's Modified Eagle's Medium (DMEM) (Gibco #30-2002), supplemented with 10% fetal bovine serum (FBS) (Gibco #10437-028), 100 U/ml of penicillin, and 100 μg/ml of streptomycin (Gibco #15140-122) in a 37ºC humidified 5% CO_2 incubator. For cell passage, 80-90% confluent cells were washed with phosphate buffered saline (PBS) and trypsinised with 1X Trypsin-EDTA (0.5%) (Gibco #15400054) for 5 minutes. The cells were split at 1:10 subcultivation ratio for regular maintenance and the complete medium was renewed every 2 to 3 times/week. The cells were counted by using the Nexcelom, Cellometer Auto T4 Plus automated cell counting system. To explore the Lgr5 regulation mediated by IL-18 signaling, CMT93 cells were treated with or without 100 ng/ml recombinant mouse IL-18 protein (R&D #9139-IL) or 5 μM MK-2206 (Santa Cruz #1032350-13-2) for 24 h.

Silencing the Lgr5 expression by small interfering RNA (siRNA)

To decrease the protein expression, recombinant lentiviruse encoding siRNAs specific to the gene of interest were purchased from the RNAi Core, Academia Sinica, Taiwan. The target sequence for mouse *Lgr5* (NM_010195) siRNA is 5'-CCCATGACTGAAAGCTGTCAT-3' (Clone ID: TRCN0000338137) or 5'-ATGACTCAATGGGTGTATTTA-3' (Clone ID: TRCN0000338078). The above-mentioned sequences were respectively cloned into the EcoRI site of the Lentiviral vector pLKO.1-puro. To produce recombinant lentiviruses, HEK 293T cells were co-transfected with vector alone or pLKO.1-siRNA insert (2.5 μg), pCMV-ΔR8.91 (2.25 μg) and pMD.G (0.25 μg) by Transfection Reagent (TransIT®-LT1, Mirus#MIR 2300) in a 6 cm dish. After 72 h, the medium was harvested, aliquoted and stored at −80 °C. To silence the gene in CMT93 cells, the cells were infected with recombinant lentiviruses encoding gene-specific siRNAs with a MOI>6 in the presence of 8 μg/ml polybrene (Sigma-Aldrich #28728-55-4) for 24 h. The cells were selected in the growth medium containing 2 μg/ml of puromycin (Gibco #A11138-03) for 5 days. The puromycin resistant cells were collected and the gene knockdown in those cells was determined by immunoblotting with a Lgr5 antibody (1:1000, Abcam #ab75850).

Epithelial crypt cell dissociation for flow cytometry analysis

Intestinal crypts were isolated and washed twice in cold PBS as described in the section of Method. The dissociated cells were stained with surface antibody markers for 25 min at 4°C and later resuspended in BD Fixation/Permeabilization solution (BD #554714) for 30 min to permeabilize the cells for intracellular staining. The cells were washed and incubated with 1X perm/wash buffer (BD #554723) containing antibodies for intracellular staining at room temperature for 30 min in dark. The cells were again washed with 1X perm/wash buffer and then analyzed by flow cytometry. For Paneth cell analysis, the cell were gated as the $CD24^{+/high}$ $Lysozyme^{+}$ subset. For Goblet cell analysis, the cells were gated as the $c\text{-}kit^{+}Muc2^{+}$, $CD24^{-/low}$ $Muc2^{+}$, or $CD24^{-/low}$ $UEA1^{+}$ subset (Ulex europaeus agglutinin-1, UEA1-FITC from Sigma-Aldrich #L9006).

Paneth cells sorting and stimulation

Intestinal crypts were isolated and washed twice in cold PBS as described in the section of Method. The dissociated cells were pooled from individual half small intestines of 3 wild-type mice and stained with surface marker (EpCAM, CD45, CD24, c-kit, and CD66a) for 30 min at 4°C. The Paneth cells staining panel was following Rothenberg et al. (Gastroenterology 142: 1195, 2012) with some modifications. The sorted Paneth cells ($EpCAM^{+}CD45^{-}CD24^{+}SSC^{+}c\text{-}kit^{+}CD66a^{-}$) were collected by BD FACSAria IIIu with BD FASCDiva software v6.2. The absolute number of sorted Paneth cells was around 50,000 cells, pooled from 3 wild-type mice. The sorted Paneth cells were stimulated with 100 ng/ml of IL-18 for 8 hr in WNR growth medium. After that, the cells were mixed with 0.1 ml TRIzol Reagent (Ambion #15596018) for RNA extraction and quantitative real-time PCR analysis.

Ileum fragment and fresh ileum crypt stimulation

Mice (8-12 weeks old) used in the experiments were euthanized with CO_2 and the abdomen was cut open to separate the small intestine. After cleaning and cutting longitudinally, ileums were cut into 1-2 mm pieces, transferred to a 24-well plate in complete RPMI buffer containing 10 % fetal bovine serum (FBS), 100 U/ml of penicillin, 100 µg/ml of streptomycin, and 10 µl/ml Gentamicin in a 37°C humidified 5% CO_2 incubator, and stimulated with 100 ng/ml of IL-22 or 100 ng/ml of IL-18 for 8 hr. Intestinal crypts were isolated and washed twice in cold PBS as described in the section of Method. The dissociated crypts were transferred to a 24-well plate in cRPMI buffer and stimulated with 100 ng/ml of IL-22 or 100 ng/ml of IL-18 for 8 hours in a 37 °C humidified 5% CO_2 incubator. The fragments or dissociated crypts were mixed with TRIzol Reagent for RNA extraction and quantitative real-time PCR analysis.

Isolation and stimulation of mesenteric lymph node (mLN) cells

Mice (8-12 weeks old) used in the experiments were euthanized with CO_2 and the abdomen was cut open to separate the whole gut (small intestine and colon). The mesenteric lymph nodes were isolated and processed into single cell suspension by gently passing through a 70 µm cell strainer using the plunger of a 3 ml syringe. The cell strainer was washed with 30 ml of cold harvest media (2% FBS in HBSS) into a 50 ml tube. mLN cells were centrifuged at 350 g for 5 min at 4°C and resuspended in complete RPMI medium. Cells were then split into two aliquots, for unstimulated or stimulated culture, in a V-bottom 96-well plate for flow cytometry analysis. After 24 hr stimulation of IL-12 (Biolegend #577002), IL-18 (Biolegend #767004), or the combination of IL-12/IL-18, the cells were briefly resuspended by pipetting before spinning down at 1,500 rpm for 5 min at 4°C and washed once with staining buffer (2% FBS in 1X cold PBS). 50 µl staining buffer containing the blocking antibody (anti-CD16/32) was added to cells for 10 min before surface staining for 20 min in dark at 4°C. For intracellular staining, the cells were fixed and permeabilized in BD Fixation/Permeabilization Solution (BD, #554714) for 30 min at 4°C, washed once with 200 µl of BD Perm Wash Buffer (BD, #554714) followed by centrifugation at 1,800 rpm, 6 min at 4°C. The cells were suspended with intracellular staining antibody in 1X Wash Buffer and stained for 30 min in dark at 4°C. After washing in staining buffer, the cells were resuspended in 200 µl staining buffer and immediately analyzed by BD LSR-II flow cytometer.

RNA extraction from the cultured organoids

To extract the RNA, the organoid pellet was lyzed in 1 ml of TRIzol Reagent (Ambion #15596018) and the homogenate was incubated for 5 min at room temperature. About 0.2 ml of chloroform was added to the homogenate, the samples were vortexed vigorously and incubated at room temperature for 15 min with occasional vortex and later centrifuged at 12,000 g for 15 min at 4°C to extract the RNA. The aqueous phase was transferred into a new tube, mixed with isopropanol at 1:1 ratio and incubated at room temperature for 10 min to precipitate the RNA. The samples were centrifuged at 12,000 g for 15 min at 4°C and the supernatant was discarded. The RNA pellet was washed with 1 ml of 75% ethanol and centrifuged at 7,500 g for 5 min at 4°C for three times and the resulting pellet was dissolved in DEPC-H_2O and RNA concentration was measured.

cDNA synthesis and quantitative real-time PCR

Mice were euthanized and the intestines were isolated, longitudinally opened, and immediately washed with ice-cold PBS. Crypts isolation and organoid culture were performed as described above. A small fragment of the intestine was cut horizontally for RNA extraction in the TRIzol Reagent (Ambion #15596018). Total RNA were reverse transcribed by using iScript™ cDNA

Synthesis Kit (Bio-Rad #1708891). cDNA was amplified by using Power SYBR® Green PCR Master Mix (Applied Biosystems #4367659) according to the provided instruction. Data were collected by ABI StepOnePlus Real-Time PCR Systems with ABI StepOne Software (v2.3). The house-keeping gene *l32* was used as an internal control for normalization.

Immunofluorescence staining of tissue sections

Mouse tissues were fixed in freshly prepared 4% formaldehyde in PBS overnight at 4°C, depending upon the experiment, the tissue was either dehydrated in ethanol and embedded in paraffin or dehydrated in 30% sucrose in PBS and frozen in OCT compound (FSC 22 Frozen Section Media, Leica Biosystems #3801480). Paraffin tissue slices or cryosections (6 µm in thickness) were sectioned by the Pathology Core at Institute of Biomedical Sciences, Academia Sinica, and mounted on positively charged glass slides. Paraffin sections were dewaxed and heat-mediated antigen retrieval was performed by incubation in either 10 mM sodium citrate buffer pH 6.0 for 10 min or 1 mM EDTA pH 8.0 for 5 min at a sub-boiling temperature. Slides were then blocked with blocking buffer for 1 hr and subjected to sequential incubation with primary antibodies at 4°C overnight and fluorophore-conjugated secondary antibodies at room temperature for 2 hr. When applicable the slides were double-stained, between each incubation step, the slides were washed with PBS three times for 5 min. The antibodies were diluted in antibody dilution buffer. For cell death detection, the tissues was stained with *In Situ* Cell Death Detection Kit (TMR red, Roche #12156792910) for a TUNEL assay. To visualize the nucleus, slides were counterstained with Hoechst 33342 (1 µg/ml in PBS) for 10 min and cover slips were mounted on the slides by using ProLong Gold Antifade Mountant. Confocal images were obtained with a Carl Zeiss LSM 700 stage imaging system under a 20x, 40x or 100x oil-immersion objectives with ZEN 2011 software.

Generation of lentiviral vectors expressing mouse Noggin and R-spondin-1

The mouse Noggin and R-spondin-1 cDNA expressing vectors (pCMV6-mouse Noggin #MC209047; pCMV6-mouse R-spondin1 #MC212441) were purchased from OriGene Technologies, Inc, Rockville, MD 20850, USA. The lentiviral cDNA expression system was provided by the National RNAi Core Facility, Academia Sinica, Taiwan. The DNA fragment encoding Noggin or R-spondin-1 was amplified by polymerase chain reaction (PCR) using pCMV6-mouse Noggin and pCMV6-mouse R-spondin-1 plasmids as the template. The PCR was carried out according to the standard procedures by using the HotStarTaq *Plus* DNA Polymerase kit (Qiagen #203605): denaturing at 95°C for 30 sec, hybridizing at 60°C for 30 sec, and elongating at 72°C for 1 min. The primer sequences for Noggin were: (Forward primer) 5' GCTAGCACC**ATG**GAGCGCTGCCCCAGCCTGGG 3' and (Reverse primer) 5' AGAATTCCT**CTA**GCAGGAACACTTACACTCGG 3' and those for R-spondin-1 were

(Forward primer) 5' GCTAGCACC**ATG**CGGCTTGGGCTGTGCGTGGT 3' and (Reverse primer) 5' GAATTC**TCA**CTGTGCCCAGGTAGGTCCTACTG 3'. In the forward primers the underlined sequence is *Nhe* I restriction site and alphabets in bold phase are start codons, whereas in the reverse primers the underlined sequence is *Eco*RI restriction site and alphabets in bold phase are stop codons. The amplified PCR products were analyzed in 1% agarose gel, and visualized by ClearVision DNA Stain (PROTECH #SA-D1001). The full length cDNA fragment of Noggin was 699 base-pairs (bp), and that of R-spondin-1 was 798 bp. The respective fragments were gel eluted by using the PureLink™ Quick Gel Extraction and PCR Purification Combo Kit (Invitrogen #K220001) and cloned into pCR2.1-TOPO vector (Invitrogen #450641) and transformed into XL10-Gold Ultracompetent Cells (Agilent Technologies # 200314). Blue-white bacterial colonies were screened by digesting the plasmid DNA with *Eco*RI restriction enzyme (NEW ENGLAND BioLabs Inc. #R0101S) and positive clones were cultured in Lysogeny Broth (LB) to extract the recombinant plasmid DNA by Mini prep. The recombinant plasmids pCR2.1-TOPO-Noggin and pCR™2.1-TOPO-Rspondin-1 were double digested with *Nhe*I (NEW ENGLAND BioLabs Inc. #R0131S) first and then with *Eco*RI restriction enzymes. The Noggin and Rspondin1 cDNA target bands were purified from the gel and subsequently inserted in frame into the *Nhe*I and *Eco*RI restriction sites of the lentiviral vectors pLAS5w.Ppuro (RNAi core #C6-8-39) and pLAS3w.Pneo (RNAi core #C6-8-30) respectively and transformed into One-Shot Stbl3 competent *E. coli* cells (Invitrogen #C737303). Positive clones were selected and cultured in Terrific Broth to extract the recombinant lentiviral plasmids by using the Plasmid DNA Extraction Mini Kit (LabPrep #LPPS100). Sequences of both the cDNAs were verified by using forward primer: (1270-1288) 5' CTA GAC TCT GGC TAA CTA G 3' and reverse primer (1579-1558) 5' GTT GCT ATT ATG TCT ACT ATT C 3' for pLAS5w.Ppuro-mouse-Noggin construct, whereas for pLAS3w.Pneo-mouse-R-spondin1 construct forward primer (1904-1922) 5' CTG GTT ATT GTG CTG TCT C 3' and reverse primer (2100-2080) 5' CTA CTA TTC TTT CCC CTG CAC 3' were used.

Generation of HEK293 stable cell lines expressing mouse Noggin and R-spondin-1

To produce recombinant lentiviruses, 80-90% confluent HEK 293T cells were co-transfected with 2.5 μg of pLAS5w.Ppuro-mouse-Noggin or pLAS3w.Pneo-mouse-R-spondin-1 recombinant lentiviral plasmids, and packaging vectors pCMV-ΔR8.91 (2.25 μg), pMD.G (0.25 μg) by PolyJet™ *In Vitro* DNA Transfection Reagent (SignaGen Laboratories #SL100688) in a 6 cm dish. The medium was replaced with 3 ml complete DMEM 24 h post-transfection and after 48 h the medium was harvested, aliquoted and stored at -80°C. To establish Noggin or R-spondin-1 stably expressing HEK 293 cells, the cells were infected with recombinant lentiviruses encoding mouse Noggin or R-spondin-1 with a multiplicity of infection (MOI) >6 in the presence of 8 μg/mL polybrene (Sigma-Aldrich #28728-55-4) in complete DMEM for 24h.

The cells were rinsed by DMEM and allowed to grow in the growth medium for another 48 h. Subsequently, the cells infected with recombinant lentiviruses encoding mouse Noggin (hereafter 293-mNoggin cell line) were selected in the growth medium containing 2 μg/ml of puromycin (Gibco #A11138-03) for 1 week, whereas the cells infected with recombinant lentiviruses encoding mouse R-spondin-1 (hereafter 293-mR-spondin1 cell line) were selected in the growth medium containing 800 μg/ml Geneticin (G418 Sulfate) (Gibco #10131-035) for 14 days. The antibiotic-resistant HEK293-mNoggin or HEK293-mR-spondin-1 cells were cultured and the secretion of Noggin and R-spondin-1 proteins into the supernatant was determined by immunoblotting with anti-Noggin (1:1000, Santa Cruz #sc-25656) and anti-R-spondin-1 (1:1000, Santa Cruz #sc-49090) antibodies.

Preparation of conditioned medium for organoid culture

NR conditioned medium: After selection in antibiotics, HEK293-mNoggin and HEK293-mR-spondin-1 cells from a 10 mm cell culture dish were split at the1:5 ratio, seeded into 150 mm cell culture dishes (Corning #353025), and cultured without antibiotics in 25 ml growth medium [advanced DMEM/F-12 (Gibco #12634-010) supplemented with 10% FBS, 100 U/mL penicillin/100 μg/ml streptomycin, 50 μg/ml Gentamicin, and 2mM GlutaMAX] at 37°C humidified 5% CO_2 incubator for 4~6 days (approximately to confluency). The Noggin and R-spondin-1 conditioned medium were mixed at the 1:1 ratio and sterilized by a 0.45 um vacuum filter (Corning #430770) to produce NR conditioned medium (consisting of Noggin and R-psondin-1 proteins). A total volume of 500 ml NR conditioned medium was generated and aliquoted into 50 ml falcon tubes and freezed at -80°C.

WNR conditioned medium: The Wnt-3A glycoprotein secreting cell line L Wnt-3A (ATCC #CRL-2647) was purchased from ATCC. The cells were cultured in a 10 mm culture dish in the presence of above-mentioned growth medium containing 400 μg/ml G418 sulfate. To produce Wnt-3A conditioned medium, the cells were split at the 1:10 ratio, seeded into 150 mm culture dishes, and grown to confluency in growth medium without G418 for 6 days. The supernatant is collected as the Wnt-3A conditioned medium and it was mixed with NR conditioned medium at 1:1 ratio to produce the WNR conditioned medium. A total volume of 500 ml WNR conditioned medium was filter sterilized, aliquoted into 50 ml falcon tubes, and frozen at -80°C.
