# Supplementary material for: IL-22 initiates an IL-18-dependent epithelial response circuit to enforce intestinal host defence
Source: Nat Commun. 2022 Feb 15;13:874. doi: 10.1038/s41467-022-28478-3 (PMC8847568; doi:10.1038/s41467-022-28478-3)
Supplement: Supplementary file 3 — Reporting Summary [file 41467_2022_28478_MOESM3_ESM.pdf]

nature research

Corresponding author(s): Jr-Wen Shui

Last updated by author(s): Dec 21, 2021

Reporting Summary

Nature Research wishes to improve the reproducibility of the work that we publish. This form provides structure for consistency and transparency in reporting. For further information on Nature Research policies, see Authors & Referees and the Editorial Policy Checklist.

Statistics

For all statistical analyses, confirm that the following items are present in the figure legend, table legend, main text, or Methods section.

n/a	Confirmed	
☐	☒	The exact sample size (*n*) for each experimental group/condition, given as a discrete number and unit of measurement
☐	☒	A statement on whether measurements were taken from distinct samples or whether the same sample was measured repeatedly
☐	☒	The statistical test(s) used AND whether they are one- or two-sided<br>*Only common tests should be described solely by name; describe more complex techniques in the Methods section.*
☒	☐	A description of all covariates tested
☐	☒	A description of any assumptions or corrections, such as tests of normality and adjustment for multiple comparisons
☐	☒	A full description of the statistical parameters including central tendency (e.g. means) or other basic estimates (e.g. regression coefficient) AND variation (e.g. standard deviation) or associated estimates of uncertainty (e.g. confidence intervals)
☐	☒	For null hypothesis testing, the test statistic (e.g. *F*, *t*, *r*) with confidence intervals, effect sizes, degrees of freedom and *P* value noted<br>*Give P values as exact values whenever suitable.*
☒	☐	For Bayesian analysis, information on the choice of priors and Markov chain Monte Carlo settings
☒	☐	For hierarchical and complex designs, identification of the appropriate level for tests and full reporting of outcomes
☒	☐	Estimates of effect sizes (e.g. Cohen's *d*, Pearson's *r*), indicating how they were calculated

Our web collection on statistics for biologists contains articles on many of the points above.

Software and code

Policy information about availability of computer code

Data collection	BD FACS Diva Software (v6.1.3) (for analysis)<br>BD FACS Diva software (v6.2) (for sorting)<br>ABI StepOne Software (v2.3)<br>Zeiss LSM700 stage - Zen 2011 (SP6 64 bit Black edition) (For Confocal Imaging)<br>Leica model DMi8 - Leica Application Suite X (1.9.0.13747) (For Microscopy Imaging)<br>Nexcelom, Cellometer Auto T4 Plus (cell counting system)<br>ImageQuant LAS 4000 mini (multipurpose CCD camera system)
Data analysis	GraphPad Prism (v8.0.2)<br>FlowJo (v10.0.7)<br>Zen 2011 (SP6 64 bit Black edition) (For Confocal Image Analysis)<br>Leica Application Suite X (1.9.0.13747) (For Microscopy Image Analysis)<br>Cellometer Auto Counter (v338) (cell counting software)<br>ImageJ (1.53k)

For manuscripts utilizing custom algorithms or software that are central to the research but not yet described in published literature, software must be made available to editors/reviewers. We strongly encourage code deposition in a community repository (e.g. GitHub). See the Nature Research guidelines for submitting code & software for further information.

Data

Policy information about availability of data

All manuscripts must include a data availability statement. This statement should provide the following information, where applicable:

- Accession codes, unique identifiers, or web links for publicly available datasets
- A list of figures that have associated raw data
- A description of any restrictions on data availability

The Source Data, containing all the raw data in each figure of the main manuscript and supplementary information, as well as all uncropped Western blots and DNA gel blots, are provided with this paper. Transcription factor binding sites, illustrated in Supplementary Fig.3a, 3c, and 8c, are predicted by the open-access database JASPAR (https://jaspar.genereg.net). The PCR primers used for all ChIP assays are designed by Primer3web (https://primer3.ut.ee/).

Field-specific reporting

Please select the one below that is the best fit for your research. If you are not sure, read the appropriate sections before making your selection.

☒ Life sciences ☐ Behavioural & social sciences ☐ Ecological, evolutionary & environmental sciences

For a reference copy of the document with all sections, see nature.com/documents/nr-reporting-summary-flat.pdf

Life sciences study design

All studies must disclose on these points even when the disclosure is negative.

Sample size	Sample size for each experiment is indicated in the figure and in the Statistical analysis section. No statistical approaches were used in this study to pre-determine the sample size of experiments. We used sample size at least of three or more for each independent experiment, commonly exploited by researchers in the field. (doi:10.1016/j.immuni.2015.09.003; doi:10.1038/ni.2002; doi:10.1038/ni1271; doi:10.1038/ni.3278; doi:10.1038/nature11535)
Data exclusions	No datasets were excluded from the experiments in this study.
Replication	At least two to three independent experiments were performed and reproducible results were always obtained and calculated to achieve statistical significance.
Randomization	All cell or animal studies were randomized for group study. Age, gender-matched littermate mice were always used for animal studies.
Blinding	In general, the investigators who performed primary organoid culture or animal study were blinded for the genotypes of mice as animals were take care of and provided by other personnel in the lab. However, investigators were not blinded to the cell line study or western blot experiments as they need to arrange and load the sample based on the treatment information.

Reporting for specific materials, systems and methods

We require information from authors about some types of materials, experimental systems and methods used in many studies. Here, indicate whether each material, system or method listed is relevant to your study. If you are not sure if a list item applies to your research, read the appropriate section before selecting a response.

Materials & experimental systems

n/a	Involved in the study	
☐	☒	Antibodies
☐	☒	Eukaryotic cell lines
☒	☐	Palaeontology
☐	☒	Animals and other organisms
☒	☐	Human research participants
☒	☐	Clinical data

Methods

n/a	Involved in the study	
☒	☐	ChIP-seq
☐	☒	Flow cytometry
☒	☐	MRI-based neuroimaging

Antibodies

Antibodies used	Rabbit anti-cleaved Caspase-3 (Asp175) [5A1E] (1:1000, Cell Signaling Technology #9664)<br>Rabbit anti-Lgr5 [EPR3065Y] (1:1000, Abcam # ab75850)<br>Rabbit anit-IL18 (1:1000, Proteintech #10663-1-AP)<br>Rabbit anti-phospho-Stat3 (Tyr705) [D3A7] (1:1000, Cell Signaling Technology #9145)<br>Mouse anti-Stat3 [124H6] (1:1000, Cell Signaling Technology #9139)<br>Mouse anti-b-actin [C4] (1:1000, Santa Cruz Biotechnology #sc-47778)<br>Rabbit anti-a-tubulin (1:1000, Cell Signaling Technology #2144)

Rabbit anti-TCF4 /TCF7L2 [C48H11] (Cell Signaling Technology #2569)
Rabbit anti-Akt [N3C2] (1:2000, GeneTex #GTX121937)
Rabbit anti-phospho-Akt (Ser473) [D9E] (1:1000, Cell Signaling Technology #4060)
Rabbit anti-Noggin [FL-232] (1:1000, Santa Cruz #sc-25656)
Rabbit anti-R-spondin [C13]-R (1:1000, Santa Cruz #sc-49090)

Rabbit anti-Ki67 [SP6] (1:200, Abcam #ab16667)
Mouse anti-Lysozyme (1:100, St John's Laboratory #STJ16101308)
Rabbit anti-Lysozyme [EPR2994(2)] (1:250, Abcam #ab108508)
Rabbit anti-Olfm4 [D6Y5A] (1:200, Cell Signaling Technology #39141)
Mouse anti-E-Cadherin [4A2] (1:200, Cell Signaling Technology #14472)
Rabbit anti-Muc2 [H-300] (1:100, Santa Cruz #sc-15334)

Rat anti-CD326 (EpCAM) [G8.8] BV510 (1:400, BD Biosciences #747748)
Rat anti-CD326 (EpCAM) [G8.8] BV421 (1:200, BD Biosciences #563214)
Hamster anti-CD3e [145-2C11] FITC (1:200, eBioscience #11-0031-85)
Rat anti-CD3 [17A2] BV786 (1:200, BD Biosciences #564010)
Rat anti-CD45 [30-F11] Alexa Fluor 700 (1:400, eBioscience #56-0451-82)
Rat anti-CD45 [30-F11] APC (1:400, Biolegend #103112)
Rat anti-CD4 [RM4-5] Brilliant Violet 785 (1:200, Biolegend #100552)
Rat anti-CD4 [RM4-5] eFluor 450 (1:200, eBioscience #48-0042-82)
Rat anti-CD8a [53-6.7] Brilliant Violet 650 (1:200, Biolegend #100742)
Rat anti-CD11b [M1/70] PE (1:200, Biolegend #101208)
Rat anti-Ly-6G/Ly-6C (Gr1) [RB6-8C5] PE-Cyanine7 (1:400, eBioscience #25-5931-82)
Mouse anti-NK1.1 [PK136] Alexa Fluor 700 (1:200, eBioscience #56-5941-82)
Rat anti-CD90.2 (Thy1.2) [30-H12] PerCP-eFluor 710 (1:400, eBioscience #46-0903-82)
Rat anti-CD90.2 (Thy1.2) [53-2.1] V500 (1:400, BD Biosciences #561616)
Rat anti-CD24 [M1/69] APC-eFluor 780 (1:200, eBioscience #47-0242-82)
Rat anti-CD24 [M1/69] BV605 (1:400, BD Bioscience #563060)

Rat anti-IFNg [XMG1.2] PE-CF594 (1:400, BD Biosciences #562303)
Mouse anti-IL-18 [12E7.1] unconjugated (1:200, sigma #04-1585)
Rat anti-Ki-67 [SolA15] PE-Cyanine7 (1:400, eBioscience #25-5698-82)
Rat anti-Lgr5/GPR49 [#803420] PE (1:400, R&D # FAB8240P)
Rabbit anti-active Caspase-3 [C92-605] Alexa Fluor-647 (5 ul/test, BD Biosciences #560626)
Rat anti-IL-22Ra1 [#496514] APC (1:20, R&D #FAB42941A)
Mouse anti-IL-18R1 [P3TUNYA] PE (1:200, eBioscience #12-5183-82)
Rabbit anti-Lysozyme FITC (1:200, Dako #F0372)
Rat anti-c-kit [ACK2] PE/Dazzle 594 (1:200, Biolegend #135128)
Rat anti-c-kit [2B8] APC/Cyanine7 (1:200, Biolegend # 105826)
Mouse anti-CD66a [CC1] APC (1:200, eBioscience #17-0661-80)

Secondary antibodies:
Goat anti-mouse IgG HRP (1:10000, Jackson ImmunoResearch #115-035-003)
Goat anti-rabbit IgG HRP (1:10000, Jackson ImmunoResearch #111-035-003)
Goat anti-mouse Alexa Fluor-594 (Cell Signaling Technology #8890)
Goat anti-rabbit Alexa Fluor-488 (Cell Signaling Technology #4412)
Goat anti-rabbit Alexa Fluor-594 (Cell Signaling Technology #8889)
Goat anti-rat Alexa Fluor-488 (Cell Signaling Technology #4416)
Goat anti-rat Alexa Fluor-594 (Invitrogen #A-11007)

Validation

Validation statements available from manufacturers:
Rabbit anti-cleaved Caspase-3 (Asp175) (https://www.cellsignal.de/products/primary-antibodies/cleaved-caspase-3-asp175-5a1e-rabbit-mab/9664?Ntk=Products&Ntt=9664),
Rabbit anti-Lgr5 [EPR3065Y] (https://www.abcam.com/lgr5-antibody-epr3065y-ab75850.html),
Rabbit anit-IL-18 (https://www.ptglab.com/products/IL18-Antibody-10663-1-AP.htm),
Rabbit anti-phospho-Stat3 (Tyr705) (https://www.cellsignal.com/products/primary-antibodies/phospho-stat3-tyr705-d3a7-xp-rabbit-mab/9145?Ntk=Products&Ntt=9145),
Mouse anti-Stat3 (https://www.cellsignal.com/products/primary-antibodies/stat3-124h6-mouse-mab/9139?Ntk=Products&Ntt=9139),
Mouse anti-b-actin (https://www.scbt.com/p/beta-actin-antibody-c4),
Rabbit anti-a-tubulin (https://www.cellsignal.com/products/primary-antibodies/a-tubulin-antibody/2144),
Rabbit anti-TCF4 /TCF7L2 (https://www.cellsignal.com/products/primary-antibodies/tcf4-tcf7l2-c48h11-rabbit-mab/2569?Ntk=Products&Ntt=2569),
Rabbit anti-Akt [N3C2] (https://www.genetex.com/Product/Detail/AKT-antibody-N3C2-Internal/GTX121937),
Rabbit anti-phospho-Akt (Ser473) [D9E] (https://www.cellsignal.com/products/primary-antibodies/phospho-akt-ser473-d9e-xp-rabbit-mab/4060),
Rabbit anti-Noggin [FL-232] (https://www.scbt.com/p/noggin-antibody-fl-232),
Rabbit anti-R-spondin [C13]-R (https://datasheets.scbt.com/sc-49090.pdf),
Rabbit anti-Ki67 (https://www.abcam.com/ki67-antibody-sp6-ab16667.html),
Mouse anti-Lysozyme (https://www.stjohnslabs.com/anti-lysozyme-antibody-stj16101308.html),
Rabbit anti-Lysozyme [EPR2994(2)] (https://www.abcam.com/lysozyme-antibody-epr29942-ab108508.html),
Rabbit anti-Olfm4 [D6Y5A] (https://www.cellsignal.com/products/primary-antibodies/olfm4-d6y5a-xp-rabbit-mab-mouse-specific/39141?Ntk=Products&Ntt=39141),
Rabbit anti-Muc2 [H-300] (https://www.scbt.com/p/mucin-2-antibody-h-300?productCanUrl=mucin-2-antibody-

h-300&_requestid=1473876),
Mouse anti-E-Cadherin [4A2] (https://www.cellsignal.com/products/primary-antibodies/e-cadherin-4a2-mouse-mab/14472),
Rat anti-CD326 (EpCAM) [G8.8] BV510 (https://www.bdbiosciences.com/us/applications/research/stem-cell-research/cancer-research/mouse/bv510-rat-anti-mouse-cd326-g88/p/747748),
Rat anti-CD326 (EpCAM) [G8.8] BV421 (https://www.bdbiosciences.com/en-us/products/reagents/flow-cytometry-reagents/research-reagents/single-color-antibodies-ruo/bv421-rat-anti-mouse-cd326.563214),
Hamster anti-CD3e [145-2C11] FITC (https://www.thermofisher.com/antibody/product/CD3e-Antibody-clone-145-2C11-Monoclonal/11-0031-82),
Rat anti-CD3 [17A2] BV786 (https://www.bdbiosciences.com/eu/applications/research/t-cell-immunology/th-1-cells/surface-markers/human/bv786-rat-anti-mouse-cd3-molecular-complex-17a2/p/564010),
Rat anti-CD45 [30-F11] Alexa Fluor 700 (https://www.thermofisher.com/antibody/product/CD45-Antibody-clone-30-F11-Monoclonal/56-0451-82),
Rat anti-CD45 [30-F11] APC (https://www.biolegend.com/en-us/products/apc-anti-mouse-cd45-antibody-97),
Rat anti-CD4 [RM4-5] Brilliant Violet 785 (https://www.biolegend.com/en-us/products/brilliant-violet-785-anti-mouse-cd4-antibody-7954),
Rat anti-CD4 [RM4-5] eFluor 450 (https://www.thermofisher.com/antibody/product/CD4-Antibody-clone-RM4-5-Monoclonal/48-0042-82),
Rat anti-CD8a [53-6.7] Brilliant Violet 650 (https://www.biolegend.com/en-us/products/brilliant-violet-650-anti-mouse-cd8a-antibody-7635),
Rat anti-CD11b [M1/70] PE (https://www.biolegend.com/en-us/products/pe-anti-mouse-human-cd11b-antibody-349),
Rat anti-Ly-6G/Ly-6C (Gr1) [RB6-8C5] PE-Cyanine7 (https://www.thermofisher.com/antibody/product/Ly-6G-Ly-6C-Antibody-clone-RB6-8C5-Monoclonal/25-5931-82),
Mouse anti-NK1.1 [PK136] Alexa Fluor 700 (https://www.thermofisher.com/antibody/product/NK1-1-Antibody-clone-PK136-Monoclonal/56-5941-82),
Rat anti-CD90.2 (Thy1.2) [30-H12] PerCP-eFluor 710 (https://www.thermofisher.com/antibody/product/CD90-2-Thy-1-2-Antibody-clone-30-H12-Monoclonal/46-0903-82),
Rat anti-CD90.2 (Thy1.2) [53-2.1] V500 (https://www.bdbiosciences.com/us/applications/research/stem-cell-research/cancer-research/mouse/v500-rat-anti-mouse-cd902-53-21/p/561616),
Rat anti-CD24 [M1/69] APC-eFluor 780 (https://www.thermofisher.com/antibody/product/CD24-Antibody-clone-M1-69-Monoclonal/47-0242-82),
Rat anti-CD24 [M1/69] BV605 (https://www.bdbiosciences.com/en-us/products/reagents/flow-cytometry-reagents/research-reagents/single-color-antibodies-ruo/bv605-rat-anti-mouse-cd24.563060),
Rabbit anti-Lysozyme FITC (https://www.agilent.com/cs/library/packageinsert/public/110550002.PDF),
Rat anti-c-kit [ACK2] PE/Dazzle 594 (https://www.biolegend.com/en-us/search-results/pe-dazzle-594-anti-mouse-cd117-c-kit-antibody-12783),
Rat anti-c-kit [2B8] APC/Cyanine7 (https://www.biolegend.com/en-us/products/apc-cyanine7-anti-mouse-cd117-c-kit-antibody-5905?GroupID=BLG4276),
Mouse anti-CD66a [CC1] APC (https://www.thermofisher.com/antibody/product/CD66a-CEACAM1-Antibody-clone-CC1-Monoclonal/17-0661-80),
Rat anti-IFNg [XMG1.2] PE-CF594 (https://www.bdbiosciences.com/us/applications/research/t-cell-immunology/th-1-cells/intracellular-markers/cytokines-and-chemokines/mouse/pe-cf594-rat-anti-mouse-ifn--xmg12/p/562303),
Mouse anti-IL-18 [12E7.1] unconjugated (https://www.sigmaaldrich.com/catalog/product/mm/041585?lang=en&region=TW),
Rat anti-Ki-67 [SolA15] PE-Cyanine7 (https://www.thermofisher.com/antibody/product/Ki-67-Antibody-clone-SolA15-Monoclonal/25-5698-82),
Rat anti-Lgr5/GPR49 [#803420] PE (https://www.rndsystems.com/products/mouse-lgr5-gpr49-pe-conjugated-antibody-803420_fab8240p),
Rabbit anti-active Caspase-3 [C92-605] Alexa Fluor-647 (https://www.bdbiosciences.com/us/applications/research/intracellular-flow/intracellular-antibodies-and-isotype-controls/anti-human-antibodies/alexa-fluor-647-rabbit-anti-active-caspase-3-c92-605/p/560626),
Rat anti-IL-22Ra1 [#496514] APC (https://www.rndsystems.com/products/mouse-il-22-ralpha1-apc-conjugated-antibody-496514_fab42941a),
Mouse anti-IL-18R1 [P3TUNYA] PE (https://www.thermofisher.com/antibody/product/CD218a-IL-18Ra-Antibody-clone-P3TUNYA-Monoclonal/12-5183-82),

Secondary antibodies:
Goat anti-mouse IgG HRP (https://www.jacksonimmuno.com/catalog/products/115-035-003),
Goat anti-rabbit IgG HRP (https://www.jacksonimmuno.com/catalog/products/111-035-003),
Goat anti-mouse Alexa Fluor-594 (https://www.cellsignal.com/products/secondary-antibodies/anti-mouse-igg-h-l-f-ab-2-fragment-alexa-fluor-594-conjugate/8890),
Goat anti-rabbit Alexa Fluor-488 (https://www.cellsignal.com/products/secondary-antibodies/anti-rabbit-igg-h-l-f-ab-2-fragment-alexa-fluor-488-conjugate/4412),
Goat anti-rabbit Alexa Fluor-594 (https://www.cellsignal.com/products/secondary-antibodies/anti-rabbit-igg-h-l-f-ab-2-fragment-alexa-fluor-594-conjugate/8889),
Goat anti-rat Alexa Fluor-488 (https://www.cellsignal.com/products/secondary-antibodies/anti-rat-igg-h-l-alexa-fluor-488-conjugate/4416),
Goat anti-rat Alexa Fluor-594 (https://www.thermofisher.com/antibody/product/Goat-anti-Rat-IgG-H-L-Cross-Adsorbed-Secondary-Antibody-Polyclonal/A-11007)

Eukaryotic cell lines

Policy information about cell lines

Cell line source(s)	Human epithelial, adherent colorectal adenocarcinoma cell line HT-29 (ATCC® HTB-38™)

Cell line source(s)	Mouse epithelial, adherent rectal polyploid carcinoma cell line CMT93 (ATCC® CCL-223™)<br>Human epithelial, adherent embryonic kidney cell line HEK293 (ATCC® CRL-1573™)<br>Mouse fibroblast, adherent areolar cell line L-M(TK-) transfected with a Wnt-3A expression vector (ATCC® CRL-2647™)
Authentication	All cell lines were directly obtained from ATCC with a certificate attached. And, ATCC authenticates cell lines routinely with the following tests: Cellular Morphology, Karyotyping, Cytochrome C Oxidase I (COI) Assay Testing.
Mycoplasma contamination	All cell lines or derived mutant cell lines were free of mycoplasma contamination determined by the nucleus staining or PCR amplification.
Commonly misidentified lines (See ICLAC register)	No commonly misidentified cell lines were used in the study.

Animals and other organisms

Policy information about studies involving animals; ARRIVE guidelines recommended for reporting animal research

Laboratory animals	Il-18-/- C57BL/6 mice (stock #4130), Ifng-/- C57BL/6 mice (stock #2287), Vil-Cre C57BL/6 mice (stock #4586), Stat3 flox/flox (Stat3 f/f) C57BL/6 mice (stock #16923), and Rosa26-LSL-DTA C57BL/6 mice (stock #9669) were obtained from Jackson Laboratory. Il-22-/- C57BL/6 mice were kindly provided by Dr. Wenjun Ouyang, Department of Immunology, Genentech. Defa6-Cre mice were kindly provided by Dr. Richard S. Blumberg, Department of Medicine, Brigham and Women's Hospital, Harvard Medical School. Stat3 f/f mice were bred with Defa6-Cre mice (for Paneth cell-specific) or Vil-Cre mice (for epithelium-specific), to generate conditional knockout mice. Defa6-Cre mice were bred with Rosa26-LSL-DTA mice to generate Paneth cell-deficient (PCΔ) mice. 8-12 week-old, age/gender-matched littermate mice (littermate WT vs Il-22-/- or Il-18-/-, littermate Stat3f/f vs Vil-Cre+Stat3f/f or Defa6-Cre+Stat3f/f) were used in all experiments. Wild-type controls were generated by intercrossing of heterozygous KO mice. Animals were bred separately, not co-housed, and maintained in a specific-pathogen-free (SPF) facility at a relative humidity 50±10%, 20–26°C, and in 12h dark/light cycles (08:00–20:00 light). Experiments were performed on mature animals (8-12 week-old) in both male and female mice, unless otherwise indicated.
Wild animals	The study here did not use any wild animals.
Field-collected samples	The study here did not use any samples collected from the field.
Ethics oversight	Animal care and experimental protocols (Protocol ID: 17-05-1092) have been approved by the Institutional Animal Care and Use Committee (IACUC) at the Institute of Biomedical Sciences, Academia Sinica. Ethical compliance has been observed in animal study. Dr. John T. Kung is the chair of IACUC and Ethics Committee in Academia Sinica.

Note that full information on the approval of the study protocol must also be provided in the manuscript.

Flow Cytometry

Plots

Confirm that:

☒ The axis labels state the marker and fluorochrome used (e.g. CD4-FITC).

☒ The axis scales are clearly visible. Include numbers along axes only for bottom left plot of group (a 'group' is an analysis of identical markers).

☒ All plots are contour plots with outliers or pseudocolor plots.

☒ A numerical value for number of cells or percentage (with statistics) is provided.

Methodology

Sample preparation	The biological sources of the primary cells were isolated from intestine of WT or KO mice as described in the Method section.
Instrument	BD LSR II flow cytometry, BD FACSAria IIIu sorter
Software	BD FACS Diva Software (v6.1.3) were used to collect data. FlowJo (v10.0.7) software were used to analyze data. No custom code has been deposited into a community repository.
Cell population abundance	The purity of the intestinal epithelial cells were determined by the labeling of EpCAM, a marker that is specific to epithelial cells, and CD45, a specific marker for leukocytes that do not express by epithelial cells. The purity of the WT, IL-18 KO, IL-22 KO, Defa6-Cre+Stat3 f/f, Vil-Cre+Stat3 f/f, Defa6-Cre+Rosa26-LSL-DTA epithelial cells were: 69.33 ± 4.05%, 65 ± 0.95%, 74.4 ±9.27%, 88.75 ± 3.81%, 85.37 ± 3.81%, 78.6 ± 1.41%, respectively.<br><br>The purity of ileal lamina propria cells were determined by the labeling of CD45, specific marker for leukocyte and EpCAM, a specific marker for epithelial cells that do not express by leukocytes. The purity of the WT, IL-22 KO and IL-18 KO ileal lamina propria cells were 43.52 ± 1.21%, 42.18 ± 1.72%, 54.31 ± 1.12% and , respectively.<br><br>The gating of ileal sorted Paneth cells were determined by the labeling of EpCAM, CD45, CD24, c-kit and CD66a. The purity of WT ileal Paneth cells were 0.4 ± 0.1% of total cells. Each Paneth cell sorting experiment was combined from 3 individual ileum of WT mice, and the absolute number of sorted Paneth cells was around 55,000 ± 5,000 cells.

Gating strategy

In epithelial samples gating, samples were first gated to distinguish populations of cells based on their side and forward scatter properties (SSC-A vs. FSC-A), followed by singlets (FSC-H vs. FSC-A). To define the epithelial population, the samples were gated on Live+ CD45- EpCAM+ cells. For Lysozyme, Caspase-3, Lgr5 staining, CD24, a surface marker expressed by intestinal crypts were included to enrich the population. To define lamina propria cells, the samples were gated on Live+ CD45+ EpCAM- cells. For IFNg staining, CD3, CD4, CD8, and mentioned surface markers were included to enrich and select the specific cell population.

In Paneth cells gating, samples were first gated to distinguish populations of cells based on their side and forward scatter properties (SSC-A vs. FSC-A), followed by singlets (FSC-H vs. FSC-A). To define the Paneth cells population, the samples were gated on CD45- EpCAM+ CD24+ SSC-A+ c-kit+ CD66a- cells.

☒ Tick this box to confirm that a figure exemplifying the gating strategy is provided in the Supplementary Information.
